# Integrative Analysis of Metabolomic and Transcriptomic Data Reveals Metabolic Signatures and Major Metabolic Pathways in Primary Aldosteronism

**DOI:** 10.2174/0118715303361250250119035029

**Published:** 2025-02-11

**Authors:** Xiaomei Lai, Tingting Yang, Chaoping Wei, Shuangbei Zhu, Jianling Li

**Affiliations:** 1 Department of Rehabilitation Medicine, Jiangbin Hospital of Guangxi Zhuang Autonomous Region, Nanning, Guangxi 530021, China;; 2 Department of Cardiology, Liuzhou People's Hospital, Liuzhou, Guangxi 545026, China;; 3 Department of Cardiology, First Affiliated Hospital of Guangxi Medical University, Nanning, Guangxi 530021, China;; 4 Postdoctoral Mobile Station of Guangxi Medical University, Nanning, Guangxi 530021, China

**Keywords:** Primary aldosteronism, untargeted metabolomics, transcriptomics, integration metabolism, metabolic pathway, molecular abnormalities

## Abstract

**Objective:**

Primary aldosteronism (PA) is the most common secondary hypertension. In this study, we performed the pathway enrichment analysis based on metabolomics and transcriptomic data to find the metabolic perturbations in PA, which could provide new targets for PA and further understand the biology of PA.

**Methods:**

24 PA patients and 24 healthy adults served as the control group in this study. Six participants were chosen from each group to have their peripheral blood and serum samples analyzed for omics investigations. Another eighteen participants' peripheral blood samples were selected for further validation of the RNA-sequencing results.

**Results:**

Transcriptomic analyses found 518 differentially expressed genes (DEGs), and 339 remarkably differential metabolites (DMs) were identified by untargeted metabolomics. The pathway enrichment analysis was performed by combining with the omics analysis data. We also focused on analyzing metabolic pathways that repeatedly occur and constructed possible gene-metabolic networks. A total of 5 genes and 11 metabolites showed significant changes in altered 3 lipid metabolic pathways. Furthermore, the expressions of these genes were verified by qRT-PCR.

**Conclusion:**

The combination of metabolomic and transcriptomic data can give a comprehensive picture of unique illness markers and preliminary knowledge of the molecular abnormalities underpinning PA. These findings may point to viable targets for creating treatments.

## 1 INTRODUCTION

Primary aldosteronism (PA) is regarded as the most common and potentially curable form of secondary hypertension [1]. With an excess aldosterone secretion, PA patients are more susceptible to serious cardiovascular events, renal dysfunction, stroke, metabolic syndrome, and other complications than essential hypertension patients who have similar risk profiles [2-5]. Primary aldosteronism (PA) was regarded as a rare disease that affected just 1% of people with hypertension due to an insufficient understanding of PA. With the advancement of diagnostic and detection techniques, research has revealed an increasing prevalence of PA, PA is identified as the most frequent form of secondary hypertension, accounting for 10-15% of people with hypertension [3, 6]. The prevalence rates among patients with stage 1, 2, and 3 hypertension are 11.30%, 15.70%, and 21.60%, respectively [7, 8], with an even higher incidence of around 20% among those with resistant hypertension [3, 7]. The real number of patients with PA is substantial, representing a significant economic and healthcare burden and emerging as a rapidly growing public health issue. Therefore, elucidating the molecular mechanisms of PA is crucial for the early diagnosis and treatment of PA [9, 10].

Understanding changes in metabolism is crucial because metabolic alterations often reveal the causes or outcomes of disease [11-13]. Metabolites represent the integrated endpoints of genomic, mRNA expression, and proteomic changes [14, 15], which are closely correlated with disease phenotypes [16]. Metabolomics is a complementary approach to genomics and proteomics, aiming to provide comprehensive information about dynamic metabolic responses of biological systems to pathological stimuli or genetic modifications [17]. It contributes to enhancing our understanding of the pathophysiology of diseases, identifying biomarkers for disease diagnosis and prognosis, improving diagnostic processes, developing new drugs, and optimizing treatment strategies [18-20]. Untargeted metabolomics is a comprehensive study of all metabolites within a biological system. It is a method of relative quantitative analysis to provides a thorough metabolic profile by measuring and quantifying a variety of metabolites within each sample [21]. Liquid chromatography coupled with mass spectrometry (LC-MS) has been widely applied in metabolomics in recent years [22]. Several research teams have evaluated the steroid profile of PA by using LC-MS, which not only identified new potential biomarkers for PA, but also helped in the subtyping, diagnosis, and treatment of PA [23-28]. Furthermore, it has facilitated the exploration of potential mechanisms underlying PA [29].

Although the pathogenesis of PA has not yet been elucidated, next-generation sequencing techniques have brought a new perspective to PA. KCNJ5, which encodes a K^+^ selective ion channel, was identified as the most common somatic mutation of aldosterone-producing adenoma (APA) [30]. First found by exome sequencing in 2011, the mutation is reported to elevate adrenal cell cytoplasmic Ca^+2^ concentration and thus the autonomous biosynthesis of aldosterone [30]. Recently, further somatic mutations in ATPases (ATP1A1 and ATP2B3) and ion channels (CLCN2, CACNA1H, and CACNA1D) have been found, like those in KCNJ5, which are critical for elevating CYP11B2 (aldosterone synthesis) transcription and contribute to APA tumorigenesis [1, 3]. The zona glomerulosa (ZG) associated gene DACH1 attenuated aldosterone secretion and activated the canonical Wnt and transforming growth factor-β signaling pathways [31]. The endoplasmic reticulum (ER) chaperone calmegin (CLGN) was confirmed to have a regulatory function on aldosterone synthesis by translational regulation of CYP11B2 in APA [32].

Multi-omics approaches integrate bioinformatics data from multiple levels to understand the interrelationships and joint impacts of each level in the disease process, contributing to a better understanding of their interactions and disease pathophysiological mechanisms [33, 34]. Transcriptomics unravels gene expression alterations, while metabolomics reveals metabolite shifts, and both are crucial in understanding complex disorders [35]. Moreover, precision medicine, by leveraging individual genomic and phenotypic data, aims for personalized treatments and has demonstrated enhanced therapeutic efficacy [36]. Targeted drugs have been developed based on specific gene mutations, which significantly improve patients' treatment outcomes [36]. Furthermore, molecular diagnostics, empowered by novel techniques, enable early disease detection [37].

These omics, precision medicine, and molecular diagnostics approaches are interwoven, forging a robust and all-encompassing framework that guides our exploration of the molecular intricacies within primary aldosteronism, thus enhancing the depth and clinical relevance of our study. Currently, the molecular mechanism of PA has not yet been elucidated, and multi-omics association studies of PA are rarely reported. In the present study, we sought the differentially expressed genes (DEGs) utilizing transcriptomic analysis, and untargeted metabolomic analysis was performed to reveal the metabolite changes of PA. Moreover, we focused on gene-metabolite networks in PA by integration of transcriptomic and metabolomic data to characterize specifically regulated pathways, thereby attempting to better understanding of the pathogenic mechanisms of PA.

## 2 MATERIALS AND METHODS

### 2.1 Study Participants

We included 24 patients with PA (13 males and 11 females; age: 48.9 ± 7.9 years) between March 2021 and July 2022 from the first affiliated hospital of Guangxi Medical University and 24 matched healthy adults (HA) controls (11 males and 13 females; age: 29.29 ± 4.11 years), and serum samples and peripheral blood samples were collected. Six participants from each group were selected for omics experiments analysis. Another eighteen participants were selected for subsequent verification of the RNA-sequencing results.

Our diagnostic criteria for PA were set using the current Endocrine Society guidelines [38]. PA patients were diagnosed with an increased aldosterone/renin ratio (aldosterone/renin ratio (ARR) cutoff: 57; a range of erect direct renin concentration (DRC): 2.8 to 28.5 pg/ml; a range of erect aldosterone level: 31 to 351 pg/ml)) after the discontinuation of drugs that affect the renin-aldosterone system, followed by the saline infusion test for positive confirmatory, and subsequently performing Computed Tomography (CT) scanning and adrenal vein sampling (AVS). 24 healthy adults with normal blood pressure served as the control group. The demographic and clinical characteristic data of each group were recorded and measured by professional medical personnel.

The study was approved by the Ethics Committee of the First Affiliated Hospital of Guangxi Medical University (NO:2022-KT-National Natural Science Foundation of China-284) and all participants gave informed consent. Using a standardized venipuncture process, we collected blood samples under fasting conditions in the early morning. Human peripheral blood mononuclear cells (PBMCs) from the blood sample and serum were collected, kept at -80°C, and then subjected to a subsequent experiment.

### 2.2 Untargeted LC-MS Metabolomics Analysis

Peripheral venous blood (3 - 5 ml) from the subjects was collected and placed on ice for half an hour. The upper serum was absorbed and centrifuged at 3500 rpm for 15 minutes. Six serum samples selected from the PA group and HA group were analyzed by the LC-MS system under standard protocols [39, 40]. The original data file was captured using LC-MS and transformed to mzML format using ProteoWizard software. The data then underwent peak extraction, alignment, and retention time correction by XCMS program and corrected the peak area performed by the “SVR” method. Metabolites were identified by searching the laboratory’s self-built database and integrating the public database and metDNA after filtering the peaks [39].

The data were visualized by the R platform, which was subject to univariate statistical analysis (Student’s t-test and variance multiple analysis) and multivariate statistical analysis (principal component analysis (PCA), partial least squares discriminant analysis (PLS-DA), and orthogonal partial least squares discriminant analysis (OPLS-DA)) to obtain *P* value and variable importance in the projection (VIP) value, respectively [41]. The potential differential metabolites (DMs) were screened by the false discovery rate (FDR) value less than 0.05 and VIP over 1. Additionally, we performed a Kyoto Encyclopedia of Genes and Genomes (KEGG) pathway analysis for these DMs. Metabolites were mapped to the Kyoto Encyclopedia of Genes and Genomes Database (http://www.kegg.jp/kegg/Pathway.html) [42].

### 2.3 Transcriptome Analysis

Due to one PA patient's RNA sample not meeting the criteria for transcriptomic sequencing, only 10 RNA samples corresponding to the subjects for metabolomics experiments were collected (5 from the PA group and 5 from the HA group). The obtained whole blood was combined with an equivalent amount of phosphate-buffered saline(PBS), and PBMC was separated using Ficoll-Hypaque density gradient centrifugation. The PBMC was treated with TRIzol reagent (Invitrogen, USA) at a concentration of 1×10^7^ cells per 1.5 ml to break down proteins and extract RNA. The concentration and purity of RNA sample were assessed by a NanoDrop spectrophotometer, and an Agilent 2100 Bioanalyzer (Agilent Technologies, USA) was used to measure the RNA integrity [43]. The construction of cDNA libraries was generated using an Illumina (San Diego, CA, USA) Kit and were sequenced on an Illumina sequencing platform (HiSeq™ 2500), and 125 bp/150 bp paired-end reads were produced [44, 45].

Differential genes analysis of two groups was analyzed using the DESeq2 R.package and met the following cutoff criteria: FDR value below 0.05 and |log2fold change (FC)| above 1 [46, 47]. Hierarchical clustering of DEGs was identified by using the “pheatmap” function of the R package, followed by the volcano plot and heatmap analysis [48]. We also conducted Gene Ontology (GO) enrichment analysis and KEGG pathway enrichment analysis for these DEGs by using the “clusterProfiler” and “ggplot2” R package [49, 50].

### 2.4 Integration of Metabolomic and Transcriptomic Data

The ClusterProfiler 3.5.1 was applied to identify the enrichment analysis of the DEGs. The biological pathway was constructed using KEGG (http://www.kegg.jp/kegg/ Pathway.html). The integrative analysis of DEGs-DMs was performed applying by Metaboanalyst 5.0 (https://www.metaboanalyst.ca) to identify the most relevant pathway and interactions between functionally related metabolites and genes [51].

### 2.5 Clinical Correlation Analysis

The correlation analysis was performed by GraphPad Prism 8.0.2 (San Diego, CA, USA) to calculate their Pearson correlation coefficients and *p* values. Furthermore, the biomarker analysis module in MetaboAnalyst 5.0 webserver (https://www.metaboanalyst.ca) was used to plot the receiver operating characteristic curve (ROC) analysis to explore the potential biomarkers of PA and the potential diagnostic value for DMs.

### 2.6 Validation of RNA-Sequencing Results Using Real- Time Quantitative PCR

PrimeScriptTM RT reagent Kit with gDNA Eraser kit (Takara, Japan) was used to reverse-transcribe total RNA extracted by Trizol reagent (Invitrogen, USA) into cDNA. Table **1** lists the specific primers. A real-time quantitative polymerase chain reaction (qRT-PCR) reaction mix was created using SYBR^®^ Premix Ex Taq^™^ II Kit (Takara, Japan) and amplified in triplicate using an Applied Biosystems 7500 Fast Real-Time PCR System (Thermo Fisher, USA) flowing to the standard recommendation. Values were adjusted to those of the β-actin and analyzed based on the comparative 2 -ΔΔCt method.

### 2.7 Statistical Analysis

Data were shown in mean ± standard deviation (SD). Significant analysis was conducted by SPSS22.0 statistical software (SPSS Inc. Chicago, IL, USA) with Student's t-test or Mann-Whitney U test. The diagrams were drawn on GraphPad Prism software 8.0.2 (San Diego, CA, USA). The disparities with a *P* value below 0.05 were considered statistically significant.

## 3 RESULTS

### 3.1 Clinical and Biological Characteristics of PA Patients

All patients with PA have different courses of hypertension (duration of hypertension: 87.8 ± 84.7 month), accompanied by different abnormal blood pressure (systolic blood pressure: 146.5 ± 19.6 mmHg; diastolic blood pressure: 90.5 ± 14.9 mmHg) and other important clinical and biological characteristics of PA (serum K^+^ level: 3.2 ± 0.6 mmol/L; mean of erect DRC:2.6 pg/ml; mean of erect aldosterone level: 457.9 pg/ml; mean of ARR: 378.4).

### 3.2 Metabolites Signature

**Fig. (1):**
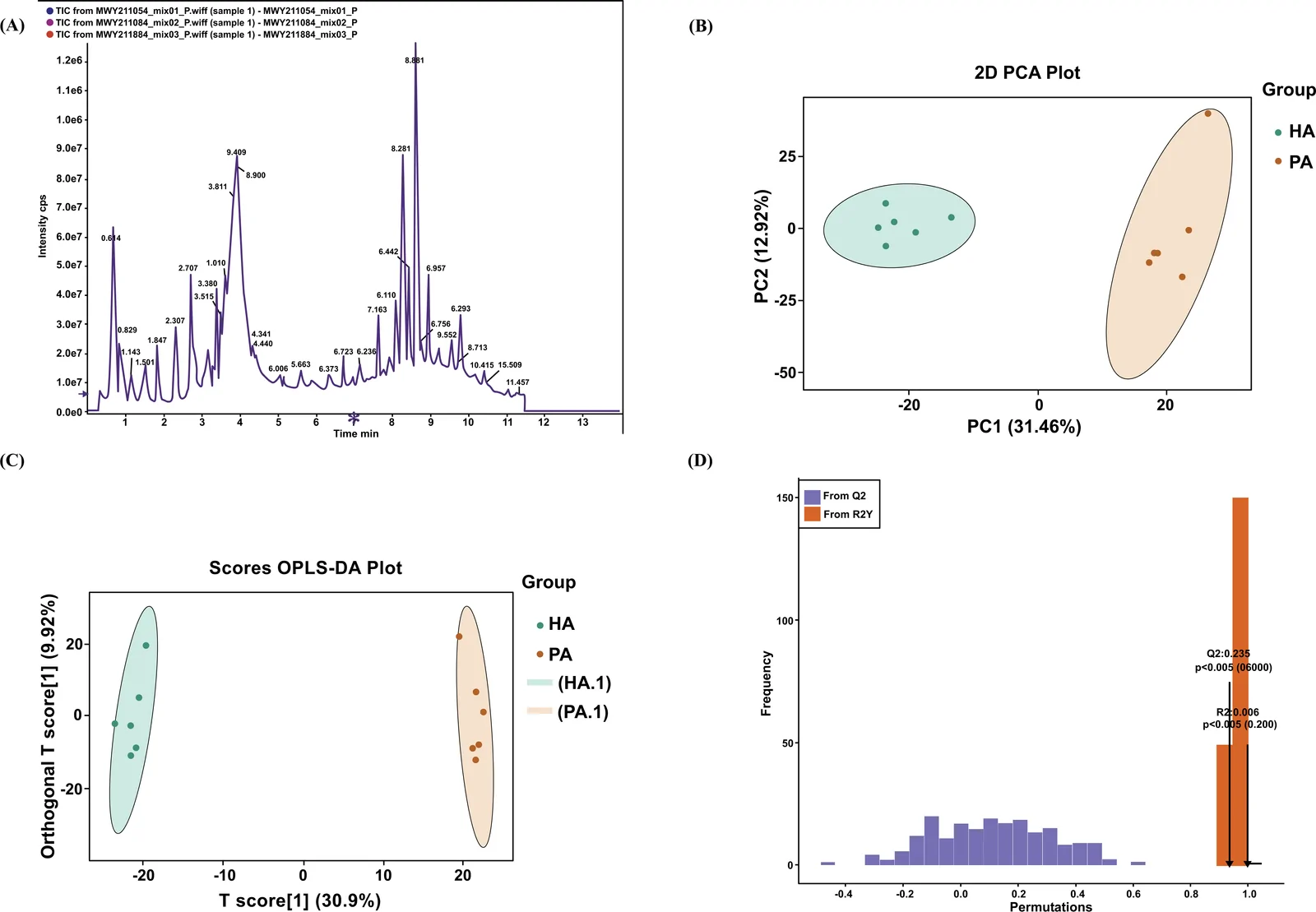
The serum untargeted metabolomic analysis of PA patients. (**A**) Overlapping TIC of QC sample; (**B**) PCA score plot of metabolic profiles for PA patients and HA; (**C**) OPLS-DA score plot of metabolic profiles for PA patients and HA; (**D**) Permutation tests with 200 iterations corresponding to the OPLS-DA model for PA patients and HA. **Abbreviations:** PA: Primary aldosteronism; HA: Healthy adults; TIC: Total ions current; QC: Quality control; PCA: Principal component analysis; OPLS-DA: Orthogonal partial least-squares discrimination analysis.

An untargeted LC-MS metabolomics detected method was carried out to identify the DMs in PA patients and HA. 1605 metabolites were detected using LC-MS in positive ion mode. The anionic mode, despite not being presented here, has been earmarked for our forthcoming research efforts. The typical total ions current (TIC) of serum samples are shown in Fig. (**1A**). PCA and OPLS-DA were used to conduct a statistical analysis of metabolite alterations. As shown in the PCA score plots and OPLS-DA score plots (Figs. **1B** and **C**), in positive ion mode, the serum samples from various groups were segregated, while samples were closely clustered in each group. The model parameters of Q2 and R2Y are 0.935 and 0.998 for Fig. (**1D**), respectively, which successfully performed the permutation test with 200 iterations.

**Fig. (2):**
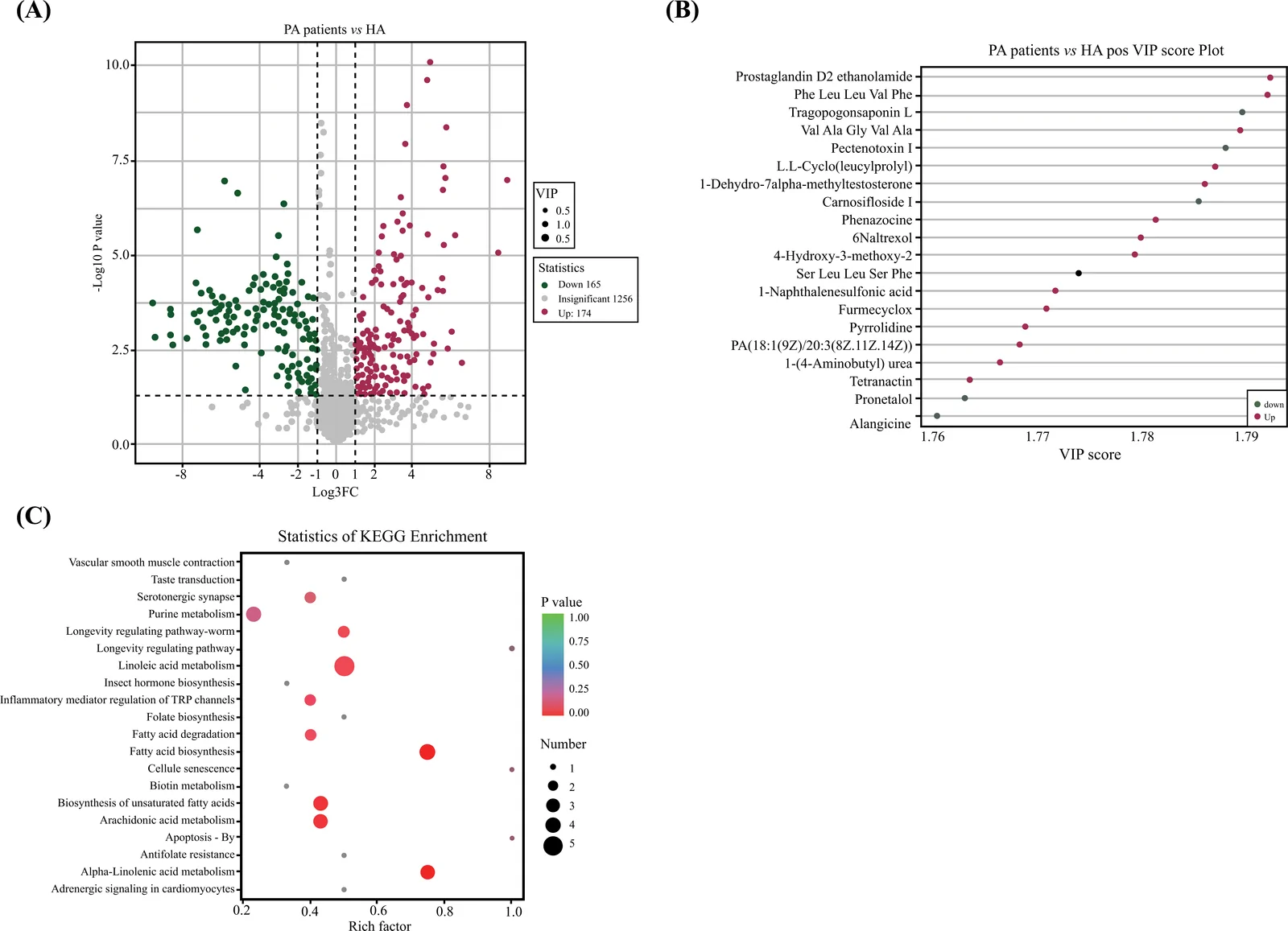
DMs in PA patients (n=6) and HA (n=6). (**A**) Volcano plots of different metabolites. The X-axis is log2 Fold-Change and the Y-axis is log10p-value. Red (up-regulated) and green (down-regulated) points indicated the different metabolites. (**B**) VIP scores of top 20 metabolites. Red (up-regulated) and green (down-regulated) points indicated the different metabolites. (**C**) KEGG pathway analysis for DMs. **Abbreviations:** PA: Primary aldosteronism; HA, Healthy adults; DMs: different metabolites; VIP: variable importance in the projection; KEGG: Kyoto encyclopedia of genes and genomes database.

The potential differential metabolites were defined as those having *P* <0.05 and VIP values > 1.0. It is evident from the volcano plot that a total of 339 DMs were identified in the PA group (Fig. **2A**), in which 174 upregulated and 165 downregulated metabolites changed significantly. The detailed DMs were listed in Supporting Information Tables **S1**. The top 20 metabolites are shown in Fig. (**2B**). To better understand the relationship between metabolic pathways and key metabolites, the DMs were mapped to pathways (Fig. **2C**). 5 metabolic pathways showed significant changes, involving linoleic acid metabolism, alpha-linolenic acid metabolism, fatty acid biosynthesis, biosynthesis of unsaturated fatty acids, arachidonic acid metabolism, which are illustrated in Table **2**.

### 3.3 Transcriptome Signature

**Fig. (3):**
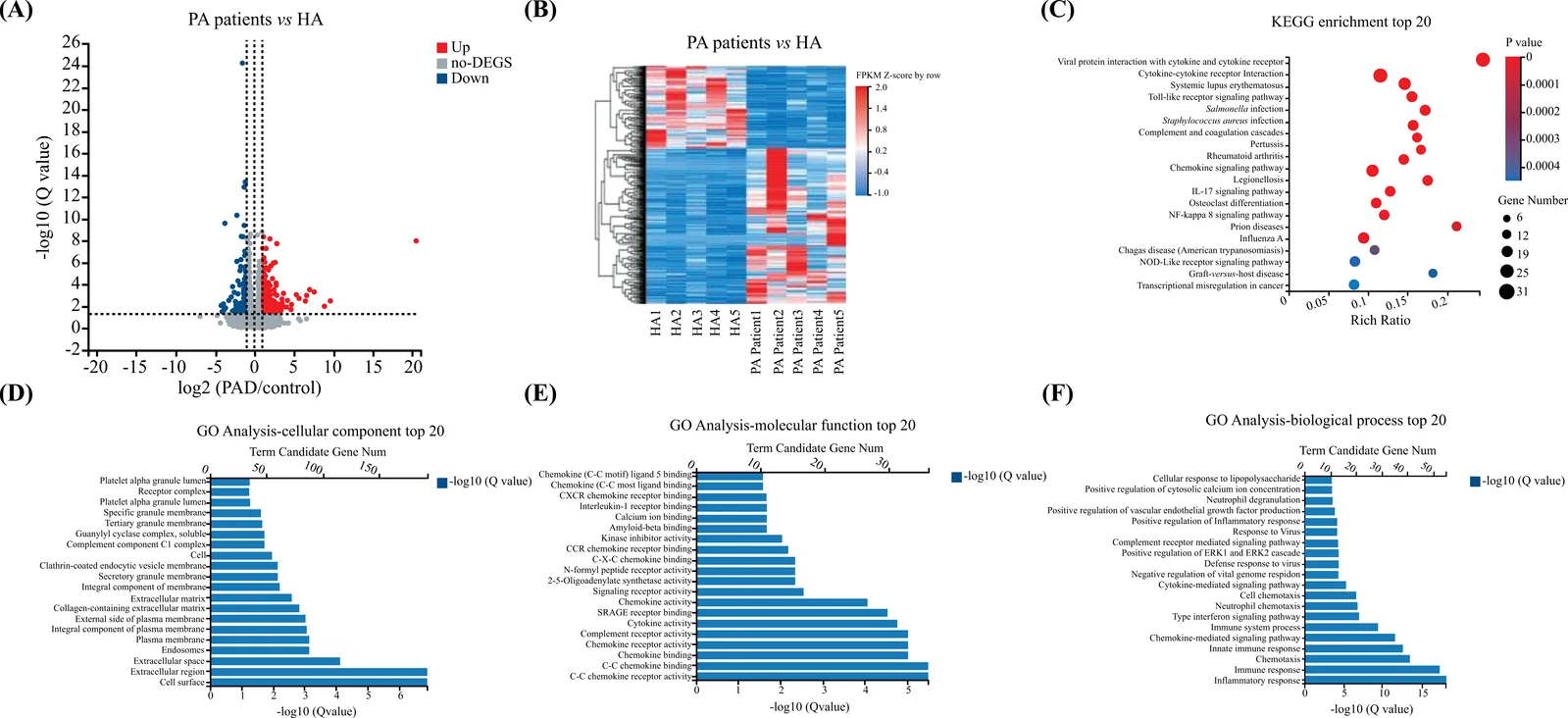
DEGs in PA patients (n=5) and HA (n=5). (**A**) Volcano plots of differentially expressed mRNAs. The X-axis is log2 Fold-Change and the Y-axis is log10p-value. Red (up-regulated) and blue (down-regulated) points indicated the differential expression of mRNA. (**B**) DEGs hierarchical clustering, each column represents a sample and every row represents a gene. The X-axis is the sample name and the Y-axis is the DEGs. Red indicates high relative expression and blue indicates low relative expression. (**C**) KEGG pathway analysis for DEGs. (**D-F**) GO analysis of DEGs that are associated with a cellular component, molecular function, and biological process. **Abbreviations:** PA: Primary aldosteronism; HA: Healthy adults; DEGs: differentially expressed genes. KEGG, Kyoto Encyclopedia of genes and genomes database. GO: Gene ontology.

Compared with healthy adults (HA) and based on the filtering condition (|log2fold change (FC)| > 1 and *P* < 0.05), a total of 518 DEGs were found in PA patients, which 339 upregulated and 179 downregulated genes changed significantly. The DEGs were shown on volcano plots (Fig. **3A**). Table **3** and Table **4** list the top 10 genes with the highest fold change in up-regulation and down-regulation, respectively. The hierarchical clustering plots suggested clear separation and consistency in the gene expression profiles of PA patients and HA (Fig. **3B**). The DEGs were listed in Supporting Information Tables **S2**.

Changes in the KEGG pathway were mainly involved in the viral protein interaction with cytokine and cytokine receptors, cytokine-cytokine receptor interaction, Toll-like receptor signaling pathway, complement and coagulation cascades, chemokine signaling pathway, IL-17 signaling pathway, NF-kappa B signaling pathway, NOD-like receptor signaling pathway (Fig. **3C**). Moreover, as shown in Fig. (**3D**-**F**), changes in Gene ontology (GO) cellular component (CC) analysis mostly enriched cell outer membranes, such as cell surface, extracellular region, extracellular spaced, and plasma membrane. Changes in the molecular function (MF) section were notably focused on receptor activity (C-C chemokine receptor activity, chemokine receptor activity, complement receptor activity, and signaling receptor activity), binding-related function (C-C chemokine binding, chemokine binding, C-X-C chemokine binding, and CCR chemokine receptor binding) and inflammatory factor activity, such as cytokine activity, chemokine activity, 2-5'-oligoadenylate synthetase activity and kinase inhibitor activity. Besides, in the biological processes (BP) section, changes were significant in inflammatory reactions (immune response, chemotaxis, inflammatory response, neutrophil chemotaxis, and cell chemotaxis) and signal transduction pathway (chemokine-mediated signaling pathway, type I interferon signaling pathway, cytokine-mediated signaling pathway, and complement receptor-mediated signaling pathway).

### 3.4 Network Analysis of Transcriptomics and Metabolomics Data

The metabolic pathway analysis of DEGs-DMs interaction was performed involving the 518 DEGs and 339 DMs with the Joint-Pathway Analysis module on Metaboanalyst 5.0. Seven metabolic pathways were identified and had statistically significant, including the pathways of arachidonic acid metabolism, nitrogen metabolism, purine metabolism, linoleic acid metabolism, alpha-linoleic acid metabolism, mucin-type O-glycan biosynthesis, glycerophospholipid metabolism as shown in Table **5** and Fig. (**4**).

**Fig. (5):**
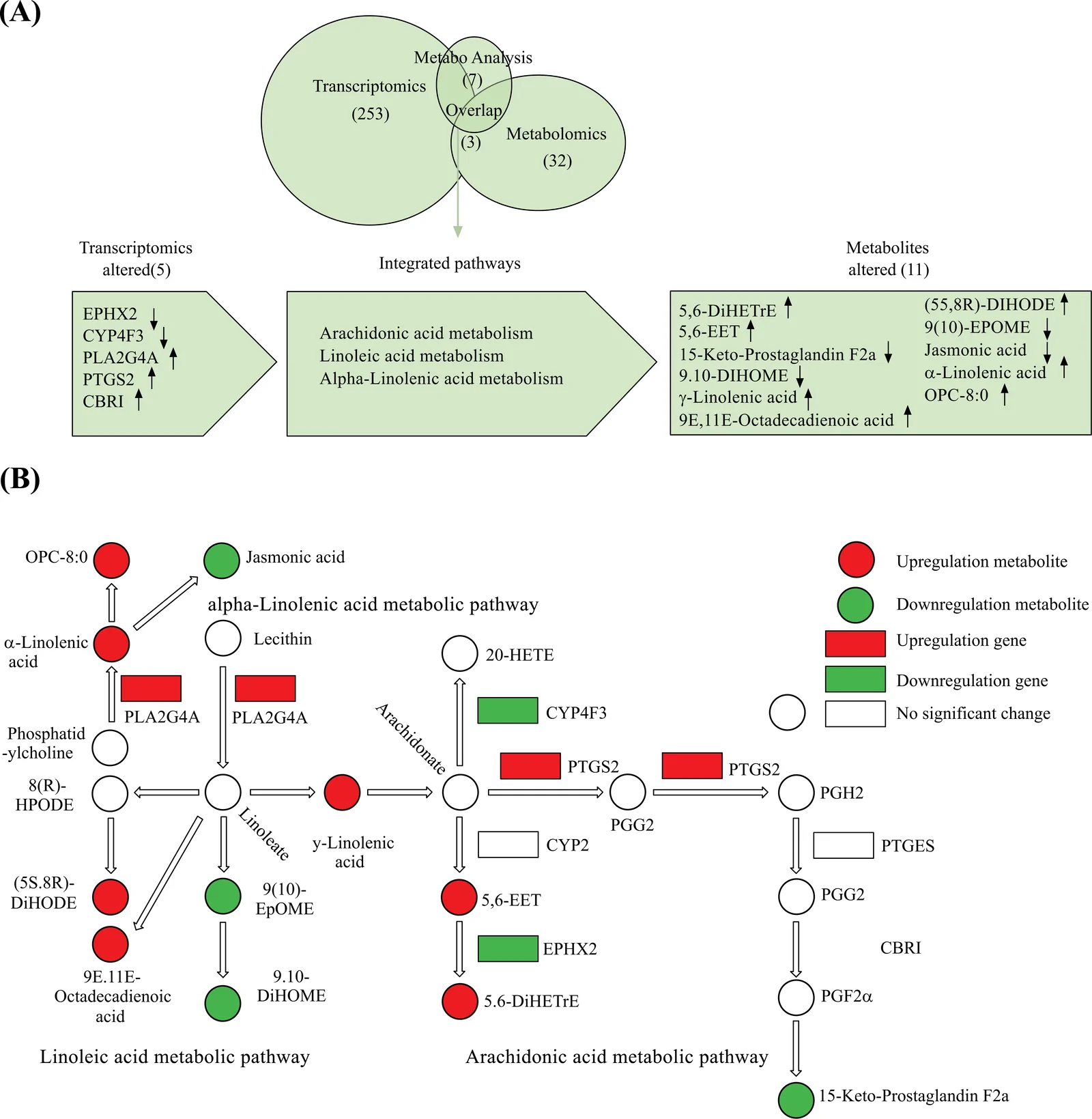
Integration of transcriptomic and metabolomic data. (**A**) Venn diagram of the metabolomic, transcriptomic and MetaboAnalysis enriched pathway. (**B**) Metabolism pathway alterations based on the metabolomic, transcriptomic and MetaboAnalysis enriched pathway data. Metabolites were shown in circles, genes were shown in boxes. Red indicated upregulation, green indicated downregulation, and white indicated no significance.

From this, combined with the enriched metabolic pathways from the transcriptomics and metabolomics data, three metabolic pathways were identified, that includes arachidonic acid metabolism, linoleic acid metabolism, and alpha-linolenic acid metabolism (Fig. **5A**). Interestingly, all three are lipid metabolism pathways. Especially, the altered genes and metabolites involved in the three metabolism pathways, along with the interconnections, are shown in Fig. (**5B**). The DEGs that relate to the integration pathways including the EPHX2 and CYP4F3 were significantly downregulated, PLA2G4A, PTGS2, and CBR1 were significantly upregulated in the PA group compared to the HA group. The differentially accumulated metabolites 15-Keto-Prostaglandin F2a, 9,10-DiHOME, 9(10)-EpOME and Jasmonic acid were significantly downregulated, while 5,6-DiHETrE, 5,6-EET, gamma-Linolenic acid(γ-Linolenic acid), 9E,11E-Octadecadienoic acid, 5S,8R-DiHODE, α-Linolenic acid and OPC-8:0 was significantly upregulated in PA patients compared to HA.

### 3.5 Clinical Correlation Analysis of Genes and Metabolites

**Fig. (6):**
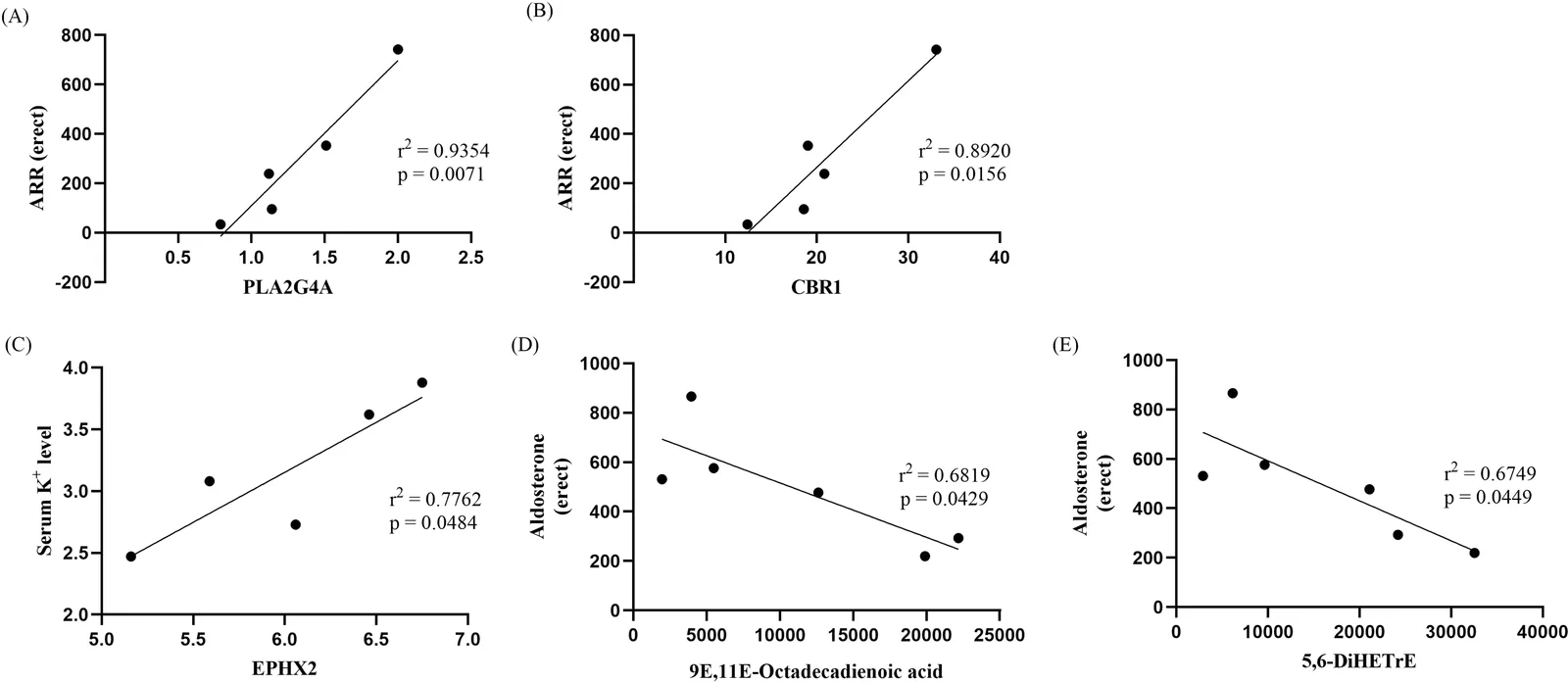
Correlation analysis. (**A**) Relationship between PLA2G4A and erect ARR. (**B**) Relationship between CBRl and erect ARR. (**C**) Relationship between EPHX2and serum K^+^ level. (**D**) Relationship between 9E,11E-0ctadecadienoic acid and erect aldosteronelevels. (**E**) Relationship between 5.6-DiHETrE and erect aldosteronelevels. ARR, aldosterone to renin ratio.

Some important parameters for diagnosing PA in clinical laboratories, such as serum K^+^level, erect aldosterone concentration, and erect aldosterone-renin ratio (ARR), are associated with the genes and metabolites identified by the integration of metabolomic and transcriptomic data (Fig. **6**). From the correlation analysis, we found a significant positive correlation between PLA2G4A and erect ARR (*P* <0.0l) as well as CBRl and erect ARR (*P*=0.0156), while serum K^+^ level was positively correlated with EPHX2 (*p* <0.05). In addition, erect aldosterone levels were negatively correlated with 5,6-DiHETrE and, 9E,11E-0ctadecadienoic acid (both *p* <0.05).

### 3.6 ROC of Metabolites

**Fig. (7):**
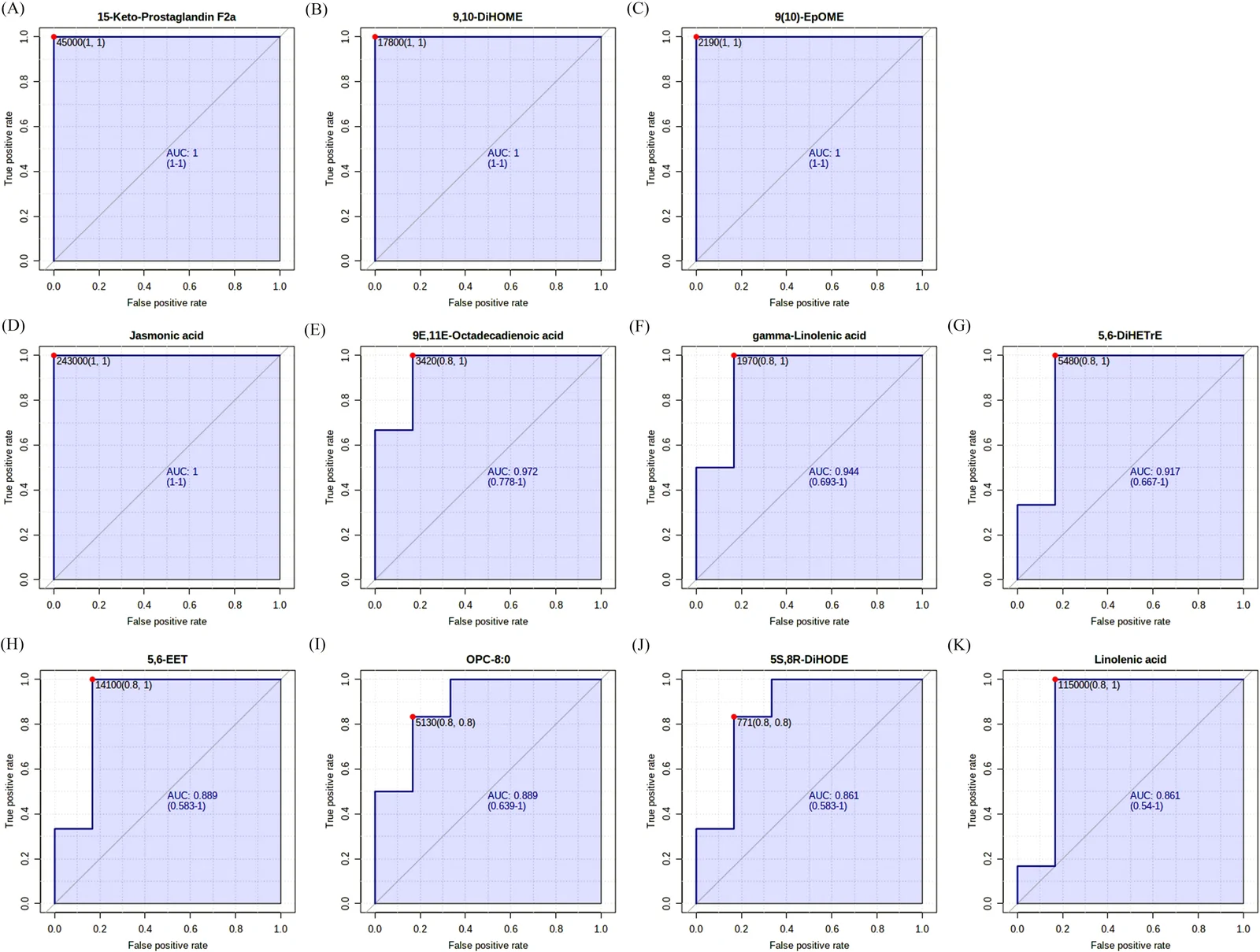
ROC analysis for diagnostic values of differential metabolites. (**A**) 15-Keto-Prostaglandin F2a. (**B**) 9,10-DiHOME. (**C**) 9(10)-EpOME. (**D**) Jasmonic acid. (**E**) 9E,11E-0ctadecadienoic acid. (**F**) gamma-Linolenic acid. (**G**) 5,6-DiHETrE. (**H**) 5,6-EET. (**I**) OPC-8:0. (**J**) 5S,8R-DiHODE. (**K**) Linolenic acid. ROC, receiver operating characteristic curve.

11 metabolites obtained from the integration of metabolomic and transcriptomic data were found to have an area under the curve (AUC) value of over 0.7 (Fig. **7**). Among them, 15-Keto-Prostaglandin F2a, 9,10-DiHOME, 9(10)-EpOME, and Jasmonic acid exhibited AUCs of 1. Moreover, 9E,11E-Octadecadienoic acid exhibited an AUC of 0.972. In addition, gamma-linolenic acid had an AUC of 0.944, 5,6-DiHETrE had an AUC of 0.917, 5,6-EET had an AUC of 0.889, OPC-8:0 had an AUC of 0.889, 5S,8R-DiHODE had an AUC of 0.861, and Linolenic acid had an AUC of 0.861. These metabolites possess excellent diagnostic power in PA, providing new clues for PA diagnosis and therapy in the future.

### 3.7 Verification of the RNA-Sequencing Results by qRT-PCR

5 genes (EPHX2, CYP4F3, PLA2G4A, PTGS2, and CBR1) implicated in arachidonic acid metabolism, linoleic acid metabolism, and alpha-linolenic acid metabolism was selected to be validated by qRT-PCR using another eighteen PA patients and HA. In general, the expression trend of most genes analyzed by qRT-PCR was highly consistent with the RNA-seq results. In comparison to HA, we discovered that the expression of EPHX was significantly down-regulated in PA patients. In addition, the expression of PLA2G4A and PTGS2 were significantly up-regulated in the PA patients compared to HA as shown in Fig. (**8**). There was no significant difference in mRNA of CYP4F3 and CBR1 between groups, but the expression trend of genes exhibited consistency with the RNA-seq results.

## 4 DISCUSSION

Primary aldosteronism (PA), which is characterized by the overproduction of aldosterone independent of the renin-angiotensin system, is the most prevalent cause of endocrine hypertension [29]. Although certain research highlighted the probable pathogenesis of PA such as germline mutations, somatic mutations, and the change of genes encoding ion channels and pumps [23], current studies on the etiology of PA remain poorly understood. The combination of transcriptomics and metabolomics analyses has not been used to study PA. Using this method, the current study builds a network consisting of genes and metabolites and focuses on the alternation of metabolic pathways, showing novel functional connections between genes and metabolites and providing fresh insights into the complicated biology of PA.

Metabolites are the cornerstone of cellular functions and participate in various enzyme-catalyzed chemical reactions, which are necessary for the operations of cells and are important bridges between genotype and phenotype [52]. We used an untargeted metabolomics approach to analyze the differential metabolites between the PA patients and HA. The metabolites are mainly associated with linoleic acid metabolism, alpha-linolenic acid metabolism, fatty acid biosynthesis, biosynthesis of unsaturated fatty acids, and arachidonic acid metabolism. A key finding from the metabolomics approach is that lipid metabolism is dramatically unregulated in PA patients. Metabolites associated with alterations in lipid metabolism are related to primary aldosteronism [53]. Diacylglycerol is responsible for regulating the expression of steroidogenic genes and ultimately producing aldosterone after stimulation of low-density lipoprotein (VLDL) [54]. Arachidonic acid, an important downstream derivative of linoleic acid, has also been demonstrated a clear correlation with aldosterone [55]. However, poor understanding currently exists regarding aldosterone's direct impact, whether in vivo or in vitro, on lipid metabolism [56, 57]. A diabetogenic effect of hypokalemia and effects of aldosterone on the insulin receptor or adipose tissue metabolism might contribute to the lipid metabolism disorders in PA [58, 59].

Consequences of the resulting inappropriately high circulating aldosterone on systemic organs include inflammation, tissue remodeling, and fibrosis, eventually leading to severe end-organ damage [60-62]. In this study, the analysis results showed that the viral protein interaction with cytokine and cytokine receptor, cytokine-cytokine receptor interaction, Toll-like receptor signaling pathway, complement and coagulation cascades, chemokine signaling pathway, IL-17 signaling pathway, NF-kappa B signaling pathway, NOD-like receptor (NLR) signaling pathway showed the most correlation with PA. Some of these signaling pathways are well- known for their important role in inflammation, immune regulation, and cell damage [63-65]. We speculate that aldosterone may produce effects of pro-oxidative, pro-fibrotic, and pro-inflammatory by increasing proinflammatory cytokines, thereby inducing systemic inflammation and fibrosis resulting in multiple systems damage and a more severe hypertensive phenotype.

We used an integrative analysis of transcriptomic and metabolomic data to provide a visualization of DEGs-DM interactions. The integrative analysis findings revealed that the DEGs-DMs were mainly clustered in the pathways of arachidonic acid metabolism, nitrogen metabolism, purine metabolism, linoleic acid metabolism, alpha-linoleic acid metabolism, mucin-type O-glycan biosynthesis, glycerophospholipid metabolism. The seven metabolic pathways were further analyzed by Venn diagram with the KEGG pathways enriched by the transcriptomics and metabolomics, and it was found that three metabolic pathways recurrence: arachidonic acid metabolism, linoleic acid metabolism, and alpha-linolenic acid metabolism. It can be seen that the changes in lipid metabolism pathways play a crucial role in PA metabolism. Increasing evidence reveals that aldosterone excess is inextricably involved in linking a higher prevalence of metabolic dyslipidemia [66, 67]. Several research have investigated the connection between aldosterone levels and metabolic syndrome, and the results show that aldosterone levels are linked to higher cholesterol, and the findings demonstrate that aldosterone levels are related to higher cholesterol, triglycerides, and lower high-density lipoprotein [68-70].

Integration of different omics data types is frequently employed to provide useful biological information at several levels, that can then elucidate potential causative changes [71, 72]. The integrative-omics analysis revealed that the metabolic pathway was mainly involved in lipid metabolism. The identified metabolites (5,6-DiHETrE, 5,6-EET, 15-Keto-Prostaglandin F2a, 9,10-DiHOME, γ-Linolenic acid, 9E,11E-Octadecadienoic acid, 5S,8R-DiHODE, 9(10)-EpOME, Jasmonic acid, Linolenic acid and OPC-8:0) were from the lipid metabolism pathways, including the arachidonic acid metabolism, linoleic acid metabolism pathways and alpha-Linolenic acid metabolism. ROC analysis indicated that the significantly changed metabolites were identified as potential biomarkers for PA, and may set the stage for future studies and clinical translation. Furthermore, the correlation analysis revealed that erectaldosterone levels were related to 5.6-DiHETrE and 9E.11E-0ctadecadienoic acid. The lipid second messengers produced by the metabolism of lipids play critical roles in regulating aldosterone synthesis and secretion [73-76]. The products of arachidonic acid metabolism were previously shown to affect aldosterone production in PA [77]. It has been demonstrated that Linoleic level and oxidized derivatives of linoleic acid are crucial for the steroidogenic response to angiotensin II in rat adrenal cells [78, 79]. However, the PA patient's lipid profile has generated debate [57, 58, 66]. The inconsistent findings might be explained by the absence of effects of insulin resistance, renal function, and obesity on lipid metabolism [57, 58]. Further studies are needed to examine potential mechanisms related to the lipid metabolism of PA.

To some extent, genetic alterations regulate metabolic pathways [80] and are essential for biological processes [45]. CD226 is linked to the proliferation and stability of vascular endothelial cells through platelet activation and plaque adhesion pathways, making it a potential novel therapeutic target for patients with isolated systolic hypertension and atherosclerotic cerebral infarction (ISH & ACI) [45]. Recent research revealed that six genes identified by bioinformatics methods play various roles in the lipid metabolism of APA suggesting lipid metabolism may regulate aldosterone secretion [81]. The study found that five genes (EPHX2, CYP4F3, PLA2G4A, PTGS2, and CBR1) were altered in the arachidonic acid metabolism pathway. Interestingly, the arachidonic acid metabolism, linoleic acid metabolism and alpha-linolenic acid metabolism pathways were both affected by PLA2G4A. PLA2G4A is considered a key gene that initiates bioactive lipid signaling and inflammatory responses by binding the calcium-dependent C2 domain [82]. EPHX2 expression was linked to metabolic processes and peroxisomal components [83], suggesting that dysregulation EPHX2 could be involved in the pathogenesis of hypercholesterolemia [84], hypertension [85], coronary heart disease [86], and metabolic reprogramming of hepatocellular carcinoma [87]. However, a direct relationship between EPHX2 and serum K^+^ level has not been well studied. A potentially functional SNP in CYP4F3 (rs4646904), which is the crucial catalyst in the oxidation of fatty acid epoxides [88], could lead to the genesis of lung cancer by affecting fatty acid metabolism [89]. The PTGS2 genes encode enzymes that are responsible for the arachidonic acid and prostaglandin biosynthesis involved in inflammation and mitogenesis [90, 91]. From the correlation analysis, we found a significant positive correlation betweenPLA2G4A and erect ARR as well as CBRl and erect ARR. To the best of our knowledge, this is the first report to correlate these genes with PA. The esterification of cholesterol plays an important role in the dissociation of amyloid-β induced signaling platforms involved in the activation of PLA2G4A in mice [66]. Previous studies have shown that CBR1 is responsible for reducing an active ketone metabolite which is identified as an inhibitor of aldosterone synthase [92]. We deduced that the up-regulation of PLA2G4A and CBRl may be related to the production of arachidonic acid and linoleic acid, which in turn affected the aldosterone generation.

## 5 LIMITATIONS

There were some limitations in our study including the relatively small sample size and the enrolled patients from a single center. It may increase the risk of type II errors and reduce the statistical power to detect more subtle differences between the groups. Besides, there is a need for further validation experiments to confirm the differential metabolites. Moreover, we only simply screened the significant genes and metabolites, and the possible mechanism of DEGs and DMs of lipid metabolism are shown in Fig. (**5**). Thus, future work will focus on the specific mechanism of particular genes and metabolite alterations both *in vivo* and *in vitro*. Furthermore, the interaction between PA and other pathways revealed in this experiment is also needed to be further investigated. In addition, future studies may consider exposure factor subgroup effects to further understand the complex relationships between exposures and disease outcomes with larger sample sizes. Despite these limitations, our investigation of PA using transcriptomics and metabolomics is the first of its type. The findings provide some light on the relationships between DEGs and DMs and help to decipher the metabolic alterations of PA.

## CONCLUSION

Our study intended to analyze alteration in pathways based on the integration of metabolomic and transcriptomic data in PA. In addition, we focused on the alteration of lipid metabolic pathways in PA. There were 5 genes and 11 metabolites being significantly altered in involving pathways, analyzing the possible regulatory relationships. This discovery may add a useful tool in research for PA and may provide new insights for the pathological and biochemical study of PA, which makes it a potential novel therapy target for PA.

## AUTHORS’ CONTRIBUTIONS

The authors confirm their contribution to the paper as follows: study conception and design: J.L.; data collection: T.Y.; analysis and interpretation of results: C.W., S.Z.; draft manuscript: X.L. All authors reviewed the results and approved the final version of the manuscript.

## Figures and Tables

**Fig. (4):**
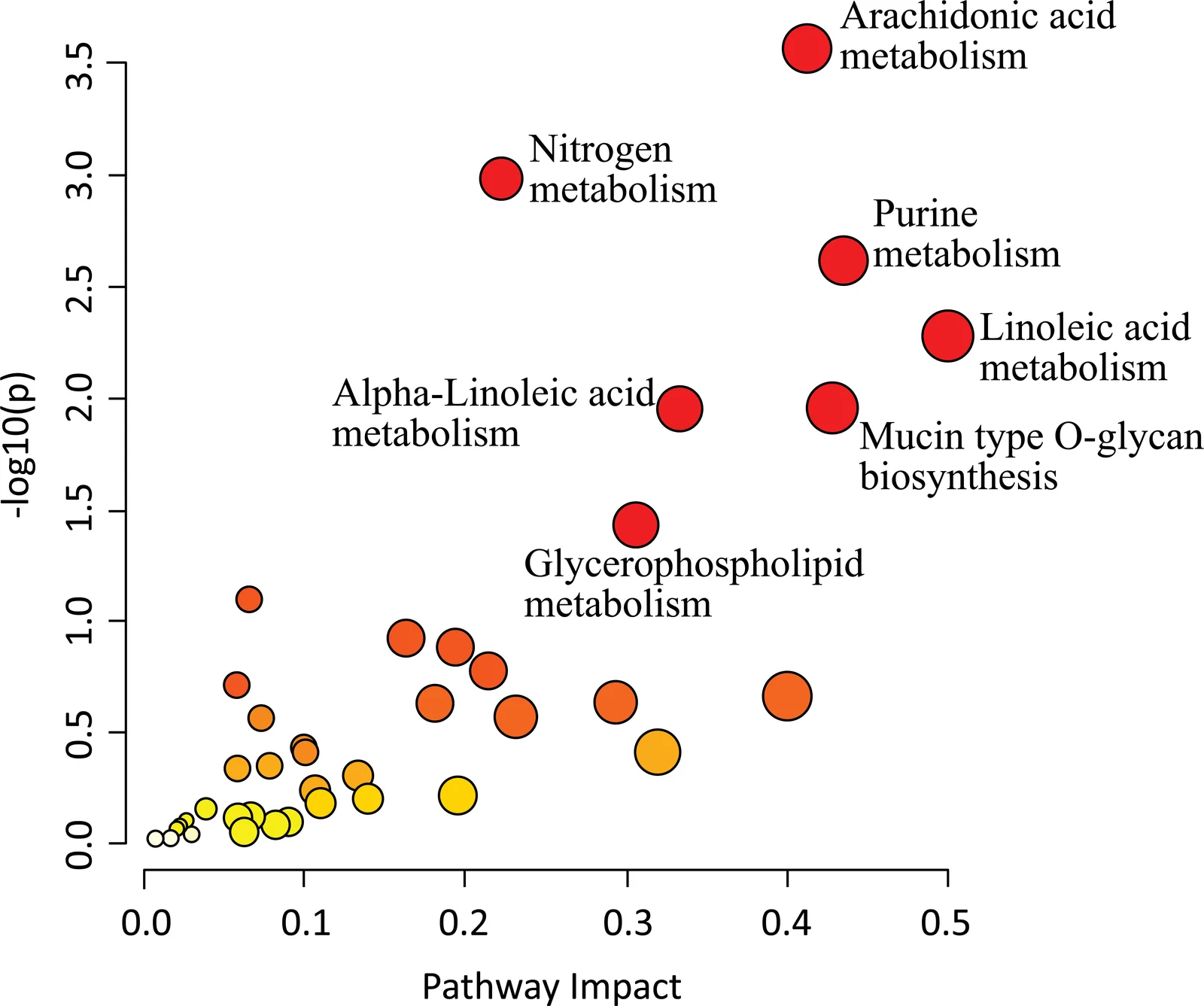
Integration of transcriptomic and metabolomic data using MetaboAnalysis. Metabolic pathway enrichment was performed with DEGs and DMs. Ordinate showed the significance and abscissa represented the impact of pathway. DEGs, differentially expressed genes; DMs, differential metabolites.

**Fig. (8):**
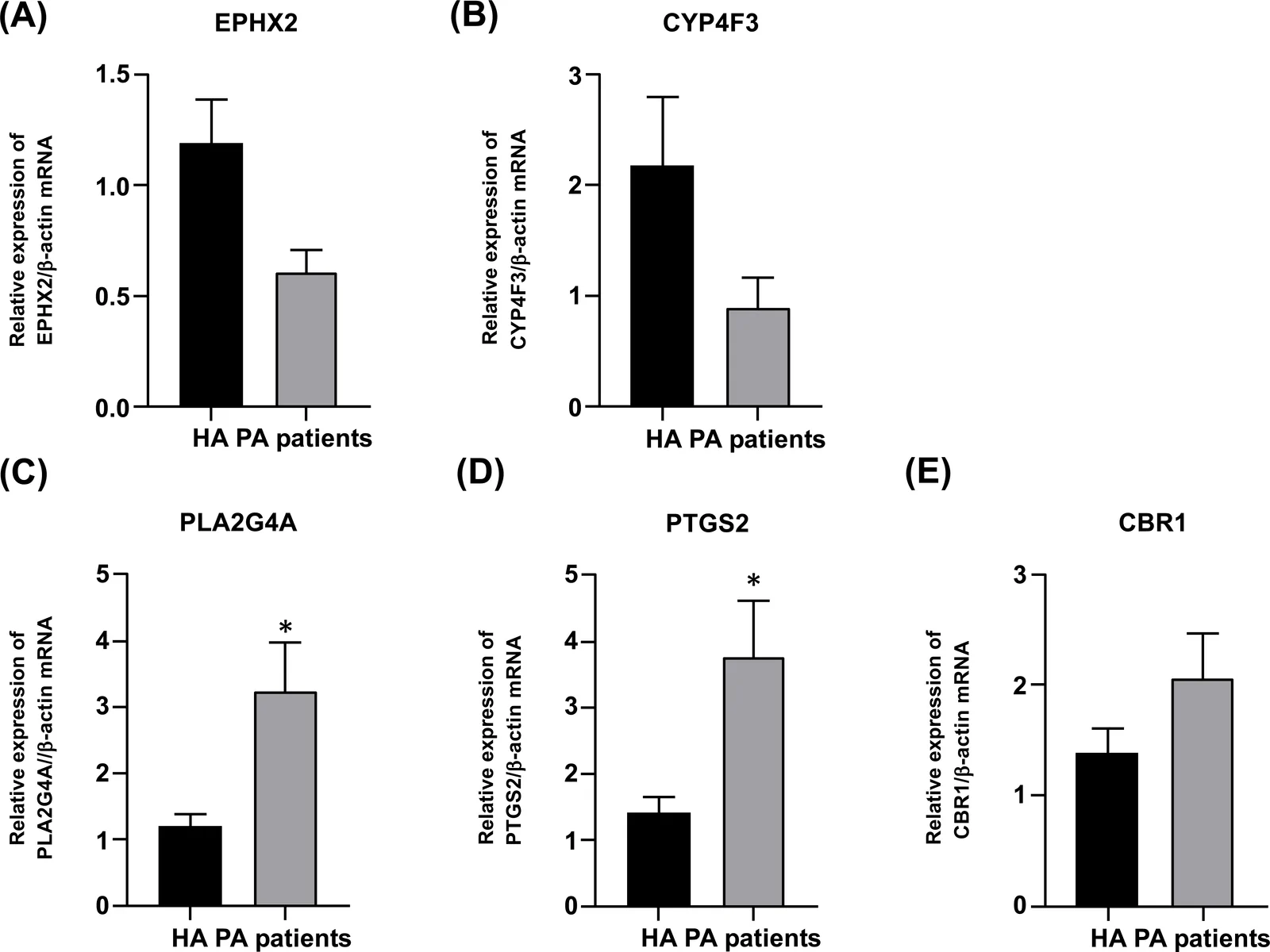
qRT-PCR validation for genes related to the arachidonic acid metabolism, linoleic acid metabolism, and alpha-linolenic acid metabolism. Data are represented as mean ± SEM (n = 18). (**A**) EPHX2. (**B**) CYP4F3. (**C**) PLA2G4A. (**D**)PTDS2. (**E**) CBR1. Significant differences from the control group were noted (*p < 0.05).

**Table 1:** Primer sequences used for qRT-PCR.

Gene	Primer Sequences (5’-3’)		Amplicon Size(bp)
EPHX2	F	TCGTCCCTTTATCTGTAAGAACCC	190
	R	AACAAAGACAGCCAGCAGCATA	
CYP4F3	F	GAGATTGAATGGGACGACCTG	154
	R	AGATAATGCCTTTGGGGATGAC	
PLA2G4A	F	GAAGGGCTGAAGGAGTGCTATG	196
	R	TTGAAAATGGTGATTCTGGGTC	
PTGS2	F	AAGATGGATGCAAAGAGGCTAGT	183
	R	GTGACAATGAGATGTGGAAAAGAAG	
CBR1	F	TGAGACCATCACTGAGGAGGAGC	120
	R	CCAATCTTCGTCACCCCGTAT	
β - actin	F	ACACTGTGCCCATCTACG	153
	R	TGTCACGCACGATTTCC	

**Abbreviations:** F, forward; R, reverse; bp, base-pairs.

**Table 2:** KEGG pathway enrichment analysis for metabolites in PA patients.

Pathway ID	KEGG Pathway	*P*-values	Metabolites
ko00591	Linoleic acid Metabolism	0.00447	9,10-DiHOME gamma-Linolenic acid 9E,11E-Octadecadienoic acid 5S,8R-DiHODE 9(10)-EpOME
ko00592	alpha-Linolenic acid metabolism	0.00749	Jasmonic acid Linolenic acid OPC-8:0
ko00061	Fatty acid biosynthesis	0.00749	Oleate Myristic acid cis-9-Hexadecenoic acid
ko01040	Biosynthesis of unsaturated fatty acids	0.04941	Oleate gamma-Linolenic acid Linolenic acid
ko00590	Arachidonic acid metabolism	0.04941	5,6-DiHETrE 5,6-EET 15-Keto-Prostaglandin F2a

**Table 3:** The most significantly up-regulated 10 genes in differentially expressed genes.

Gene Symbol	log2Fold Change	*p* Value
TBC1D3C	20.43	1.6e-11
HLA-DRB4	9.56	2.05e-4
IL1A	8.86	9.20e-4
CCL20	7.56	1.54e-5
CXCL3	6.96	7.76e-6
CXCL2	6.64	4.64e-5
F3	6.48	1.88e-4
IL1B	5.71	9.57e-5
CCL2	5.40	4.54e-5
RFPL4A	4.87	7.28e-4

**Table 4:** The most significantly down-regulated 10 genes in differentially expressed genes.

Gene Symbol	log2Fold Change	*p* Value
CA4	-4.11	8.56e-4
H2BC14	-4.02	4.99e-3
TGM3	-3.90	5.87e-4
HS3ST3A1	-3.83	3.11e-3
H1-4	-3.67	1.2e-13
CNTNAP3C	-3.63	1.70e-3
MAP7D2	-3.19	1.43e-4
H1-3	-2.85	6.51e-5
ITGAD	-2.81	5.03e-3
S100B	-2.71	3.21e-6

**Table 5:** KEGG pathway enrichment analysis for integration of transcriptomic andmetabolomic data using MetaboAnalysis.

Pathway ID	KEGG Pathway	*P*-values	Pathway impact value	-log(p)	FDR
hsa00590	Arachidonic acid metabolism	2.7408E-4	0.4125	3.5621	0.023023
hsa00910	Nitrogen metabolism	0.0010423	0.22222	2.9816	0.043813
hsa00230	Purine metabolism	0.0024412	0.43636	2.6124	0.068354
hsa00591	Linoleic acid metabolism	0.0053075	0.5	2.2751	0.11146
hsa00592	alpha-Linoleic acid metabolism	0.01134	0.33333	1.9534	0.15587
hsa00512	Mucin type O-glycan biosynthesis	0.01134	0.42857	1.9534	0.15587
hsa00564	Glycerophospholidid metabolism	0.036776	0.30588	1.4344	0.44131

## Data Availability

Data will be available upon request. The data presented in the study are included in the article. Further inquiries are available from corresponding authors upon reasonable request.

## LIST OF ABBREVIATIONS

ARR: Aldosterone/Renin Ratio
DEGs: Differentially Expressed Genes
ER: Endoplasmic Reticulum
CLGN: Chaperone Calmegin
ZG: Zona Glomerulosa
APA: Aldosterone-Producing Adenoma

## STANDARDS OF REPORTING

STROBE guidelines were followed.
