# Supplementary material for: Integrative Analysis of Metabolomic and Transcriptomic Data Reveals Metabolic Signatures and Major Metabolic Pathways in Primary Aldosteronism
Source: Endocr Metab Immune Disord Drug Targets. 2025 Feb 11;26:E18715303361250. doi: 10.2174/0118715303361250250119035029 (PMC13559962; doi:10.2174/0118715303361250250119035029)
Supplement: Supplementary file 1 [file EMIDDT-26-E18715303361250_SD1.pdf]

# Supplementary Material

## Integrative Analysis of Metabolomic and Transcriptomic Data Reveals Metabolic Signatures and Major Metabolic Pathways in Primary Aldosteronism

Xiaomei Lai[1], Tingting Yang[2], Chaoping Wei[3], Shuangbei Zhu[3] and Jianling Li[3, 4 ,*]

[1] *Department of Rehabilitation Medicine, Jiangbin Hospital of Guangxi Zhuang Autonomous Region, Nanning, Guangxi 530021, China;* [2]*Department of Cardiology, Liuzhou People's Hospital, Liuzhou, Guangxi 545026, China;* [3]*Department of Cardiology, First Affiliated Hospital of Guangxi Medical University, Nanning, Guangxi 530021, China;* [4]*Postdoctoral Mobile Station of Guangxi Medical University, Nanning, Guangxi 530021, China*

**Table S1.**

| Gene Symbol | log2 (PA /HA) |
|---|---|
| KCNE3 | 1.205308727 |
| CD300H | 1.661120671 |
| ARMCX4 | -1.00223811 |
| INSYN2B | -1.406151319 |
| GOLGA6L3 | -2.313782142 |
| LOC100287896 | 1.441385479 |
| CNTNAP3C | -3.632281453 |
| PELATON | 1.316668653 |
| TPBGL | -1.547388945 |
| TENM1 | -1.617682575 |
| LHFPL2 | 1.546850812 |
| LOC101929805 | -1.144379487 |
| TRIB1 | 1.832325544 |
| CDKN1A | 1.747174675 |
| IGSF6 | 1.31126036 |
| KCNE1B | 2.218289642 |
| CDKN1C | 1.218468444 |
| SPEG | -1.26645744 |
| CDKN2B | 1.480109127 |
| IRAG1 | 1.33360672 |
| IFITM3 | 1.824218974 |
| CDC42EP2 | 2.161676792 |
| SEMA6B | 2.613907786 |
| LOC105370092 | -1.864557811 |
| PERCC1 | -2.276786256 |
| LOC105376875 | -1.811900485 |
| LOC105377746 | -1.209613861 |
| BATF | 1.024384189 |
| KLF1 | 2.129007888 |
| CXCR6 | 1.672305058 |
| MASP2 | -1.135604955 |
| VAMP5 | 1.000392472 |
| LOC107984859 | -1.139460626 |
| LOC107985524 | -1.503809024 |
| LOC107985678 | -1.873065938 |
| HPSE | 1.104837754 |
| CD300C | 1.232744799 |
| FGL2 | 1.250068924 |
| UTS2 | 1.35328416 |
| GADD45G | -1.291480654 |
| IFI44L | 2.659304233 |
| LILRB5 | 1.719945174 |
| LILRB4 | 1.104876149 |
| LILRB3 | 1.51540862 |

| | |
|---|---|
| EEF1AKMT4 | 1.168398396 |
| ESM1 | 2.563598802 |
| CAPN11 | 2.834474958 |
| FSTL1 | 1.701164928 |
| ADAMTS7 | -1.112647024 |
| CELF3 | 2.317436688 |
| LOC112268437 | -1.275815573 |
| GALNT5 | -2.28263167 |
| EGLN3 | 1.034955318 |
| USP18 | 2.516480084 |
| TMEM106A | 1.074303654 |
| SDSL | 1.515158111 |
| KLHDC7B | 1.220821185 |
| FMNL2 | 1.040361013 |
| KLHL29 | -1.235010548 |
| NT5C3B | -1.427690491 |
| CKB | 1.610355891 |
| CISH | 1.925573898 |
| ARL11 | 1.306041175 |
| BATF2 | 2.207734867 |
| CKS2 | 1.141502558 |
| SLC16A10 | -1.270122171 |
| TDRD9 | 1.274148673 |
| CCR1 | 2.247593001 |
| CCR5 | 1.642142507 |
| CCR8 | 2.84382789 |
| CMKLR1 | 1.473687676 |
| ACER1 | -1.13244418 |
| NTN5 | -1.018846766 |
| CYP4F22 | 1.070086092 |
| CCDC163 | 1.971134647 |
| ZNF684 | 1.037967622 |
| ICA1L | -1.086992615 |
| SGPP2 | -1.006820395 |
| CD200R1 | 1.076636042 |
| FAM43A | 1.091620859 |
| SLC31A2 | 1.056586274 |
| ADORA3 | 2.297770365 |
| ADPRH | 1.544415948 |
| FRMPD2 | -2.039955399 |
| CSF1R | 1.118140915 |
| SESN3 | -1.035704675 |
| RBFOX3 | -1.03687371 |
| CIART | -1.387255264 |
| GPBAR1 | 1.549836244 |
| CTSL | 1.804217146 |
| CMTM8 | -1.287128615 |
| CX3CR1 | 2.451231665 |
| CYBB | 1.000555156 |
| CYP1B1 | 1.679801052 |
| GIMAP8 | 1.320593465 |
| ZNF483 | -1.216180483 |
| PRUNE2 | 1.683842233 |
| FAAH2 | -1.296640534 |
| ZXDB | -1.207558247 |
| USP51 | -1.058974943 |
| TMEM30B | -1.070230245 |
| FSIP1 | 2.069656629 |
| AKR1C1 | -1.365399643 |
| DDIT3 | -1.038519539 |
| CFD | 1.060833075 |
| GIMAP7 | 1.057695936 |
| GLIS3 | -1.509715207 |

| | |
|---|---|
| ADAMTS17 | -1.350991372 |
| NLRP6 | -1.11186135 |
| DUSP6 | 1.509785807 |
| TOR1A | 1.111226377 |
| EBF1 | -1.094150783 |
| TYMP | 1.493731091 |
| LPAR1 | 1.311983552 |
| EGR1 | 2.39886252 |
| EGR2 | 2.793299576 |
| EGR3 | 3.657672264 |
| MCEMP1 | 1.387739113 |
| APOBEC3A | 2.130942354 |
| TIGD1 | -1.382729557 |
| LIPH | 1.887805813 |
| SAMD14 | 1.546833528 |
| SLC16A13 | 1.127000956 |
| FAM153B | -2.107518911 |
| GAPT | 1.234908207 |
| TCTE1 | -2.039333565 |
| KHDRBS2 | -1.247253394 |
| TMEM184A | -1.291928471 |
| SLC29A1 | 1.146330521 |
| EPHX2 | -1.118758259 |
| ETV1 | 3.169403393 |
| F3 | 6.481115407 |
| SAMD9L | 1.595270634 |
| ZCCHC24 | 1.027710233 |
| UNC5B | 1.779348131 |
| MPEG1 | 1.068806314 |
| DDIAS | 1.222422751 |
| SKA3 | 1.633570565 |
| FCGR3A | 1.085297808 |
| ZBTB12 | -1.02750655 |
| ELAPOR2 | -1.068395032 |
| ADGRG3 | -2.034117205 |
| GPC4 | 1.721074759 |
| FHIT | -1.826350467 |
| RPH3A | 1.828101487 |
| MMRN1 | 1.778373257 |
| CD93 | 1.153287558 |
| MCF2L2 | -1.015727943 |
| SYNM | -1.490959368 |
| SEC14L2 | -1.341626459 |
| FPR1 | 1.380312228 |
| FPR2 | 1.927744438 |
| FPR3 | 1.975304873 |
| CLEC5A | 1.710743907 |
| PHLDA3 | 2.460020969 |
| CA14 | -1.365810962 |
| CNOT6L | -1.102863035 |
| STAC3 | 1.100398536 |
| FUT7 | 1.131965998 |
| FZD2 | 1.439977269 |
| IFI6 | 2.330153256 |
| ANKRD31 | -1.724586929 |
| MAP7D2 | -3.194328611 |
| PLCXD2 | -1.11699922 |
| CFAP45 | -1.442232303 |
| SPDEF | -2.065957346 |
| NOCT | 1.46715435 |
| DCAF12 | 1.047607411 |
| WHRN | -1.144964607 |
| TAS2R19 | -1.56201016 |

| | |
|---|---|
| IL4I1 | 1.389040228 |
| KCNG2 | 1.562361822 |
| CLEC4E | 1.422307092 |
| GBP1 | 1.827501264 |
| GCNT1 | 1.249814027 |
| MYOF | 1.31382017 |
| ITGB1BP2 | -1.041773168 |
| MYEOV | 1.013149672 |
| NAP1L5 | -1.111297415 |
| PGBD2 | 1.324110046 |
| NBEA | -1.66108237 |
| SLC39A1 | 1.135762163 |
| TOR1B | 1.284407619 |
| GLI3 | -1.329015888 |
| CCR10 | 1.066161045 |
| RBM20 | -1.756002407 |
| ANG | 1.250566631 |
| CDH20 | -1.814717517 |
| CXCR3 | 1.198513409 |
| EXOC3L1 | 2.734193956 |
| NUDT7 | 1.116027435 |
| VMO1 | 2.467798497 |
| MILR1 | 1.028558375 |
| NKPD1 | -1.360360601 |
| TMEM150B | 1.183177627 |
| RNF212 | -1.080148319 |
| GPR34 | 1.409192071 |
| ATG9B | -1.555803149 |
| C9orf47 | 1.445755985 |
| FFAR2 | 2.635304582 |
| GPT | -1.124523257 |
| COA3 | 1.051784738 |
| GRN | 1.048501331 |
| SLC43A3 | 1.127520797 |
| C1GALT1C1 | 1.155728447 |
| UBE2T | 1.189185422 |
| CXCL2 | 6.638900662 |
| CXCL3 | 6.957628841 |
| GSTM2 | -1.13137334 |
| PARVB | 1.008468401 |
| GUCY1A1 | 1.18135426 |
| GUCY1B1 | 1.074805489 |
| SAC3D1 | 1.233227873 |
| H1-3 | -2.851875371 |
| H1-4 | -3.672265954 |
| H2AC8 | -1.658015377 |
| H2BC5 | -1.136232163 |
| EHD2 | 1.516952594 |
| HLA-DRB4 | 9.558865704 |
| H2AC21 | -1.823460539 |
| APBB2 | -2.048296411 |
| HSPA1A | 1.345048565 |
| ICAM4 | 1.008321107 |
| C3orf35 | -1.151133568 |
| ANKRD34B | 2.824976629 |
| AGBL3 | -1.274055577 |
| ZC3H12B | -1.06583222 |
| EZHIP | -1.739081206 |
| IDH1 | 1.136830504 |
| CD300E | 1.290396455 |
| IFI27 | 3.571835297 |
| RFPL4A | 4.873036558 |
| IFI35 | 1.52109611 |

| | |
|---|---|
| IFIT1 | 3.017891006 |
| IFIT3 | 2.724579549 |
| NME9 | -1.387529468 |
| APOE | -1.375579817 |
| IGF1R | -1.078799777 |
| RBPMS2 | 1.673123648 |
| IGFALS | -1.438090168 |
| IGFBP7 | 1.02842683 |
| TICAM2 | 1.300824067 |
| LILRA5 | 1.375463222 |
| IL1A | 8.862876092 |
| IL1B | 5.708713908 |
| IL1RN | 3.072021866 |
| FASLG | 1.423309817 |
| IL6 | 3.599477235 |
| CXCL10 | 3.248901648 |
| IRF7 | 1.625403435 |
| ITGAD | -2.813864275 |
| C12orf42 | -1.336827 |
| TEPP | -1.685825009 |
| B3GNT8 | 1.028791516 |
| DRAXIN | 1.919535978 |
| KLHL30 | 2.074301265 |
| KRT5 | 1.882037719 |
| SUMO4 | -2.127406817 |
| RASL11A | 1.033611349 |
| C11orf96 | 3.494728142 |
| SCIMP | 1.254367579 |
| TMEM220 | -1.105014581 |
| CCL4L1 | 1.988872743 |
| CCDC103 | 2.028480942 |
| ZNF662 | -1.19537375 |
| GPR152 | -1.575039651 |
| SKOR1 | -1.355359186 |
| LAMB3 | 2.069589273 |
| LDLR | 1.405867651 |
| LGALS9 | 1.156799464 |
| SMIM10L2A | -1.408201971 |
| LMO2 | 1.022471023 |
| PRSS57 | 2.666078643 |
| SIGLEC16 | 1.507912499 |
| LMO7 | -1.074642873 |
| LRP6 | -1.247729926 |
| CYP4F3 | -1.851236161 |
| LTBP1 | 1.196293584 |
| CD180 | 1.455867347 |
| MARCKS | 1.797363284 |
| FNDC9 | -1.434154202 |
| TBC1D3C | 20.43071177 |
| MATN1 | -1.209726388 |
| MGP | -1.297225043 |
| MKI67 | 1.080285578 |
| ASCL2 | 1.08097024 |
| NR3C2 | -1.157550212 |
| MME | -1.362791705 |
| MMP1 | 4.049361182 |
| ASGR1 | 1.046811731 |
| MNDA | 1.351782506 |
| PLIN5 | -1.345538584 |
| H2BC18 | -1.8160035 |
| ODF3B | 1.257882005 |
| CALHM6 | 1.072586498 |
| MSR1 | 2.072126507 |

| | |
|---|---|
| NELL2 | -1.322059428 |
| NNAT | -1.304837872 |
| FXYD2 | -1.08020634 |
| PNP | 1.074460897 |
| ROR2 | -2.466169705 |
| OAS1 | 2.326494625 |
| OAS2 | 1.639448595 |
| OVGP1 | -1.035850601 |
| P2RY6 | 1.173648447 |
| SERPINB2 | 4.127406039 |
| CUZD1 | 1.540927893 |
| SHANK1 | -2.106541005 |
| MRPS18C | 1.136583064 |
| NDUFAF1 | 1.5831009 |
| LACTB2 | 1.150323787 |
| LEF1 | -1.006058239 |
| CLEC1B | 1.170692102 |
| TLR7 | 2.19506135 |
| TLR8 | 1.455556693 |
| MS4A4A | 2.074051823 |
| PEX5L | -1.608778716 |
| EEF1AKNMT | 1.033984434 |
| PPIL1 | 1.184257457 |
| TPPP3 | 1.076473952 |
| ASB2 | 1.09775584 |
| MSRB1 | 1.033319075 |
| VIT | 2.518403055 |
| PHKA1 | -1.284321241 |
| PLA2G4A | 1.049811483 |
| PLAG1 | -1.094379577 |
| PLCL1 | -1.086905224 |
| PLEK | 1.18838033 |
| PLK1 | 1.130531212 |
| PLXNB3 | 1.21459113 |
| GPR84 | 1.787652274 |
| SEPTIN4 | 1.274867373 |
| NLGN3 | -1.037347711 |
| MXRA8 | -1.032110661 |
| KIF1A | 4.599603077 |
| PPBP | 1.113856806 |
| XAF1 | 1.734062711 |
| EPDR1 | 1.390181121 |
| FAM20A | 3.623965665 |
| KLHL24 | -1.117691589 |
| SAMD9 | 1.192696673 |
| RPP25 | 1.567295213 |
| C14orf119 | 1.144144556 |
| CEP55 | 2.043738909 |
| CENPQ | 1.246117425 |
| GOLPH3L | 1.03678017 |
| C4orf19 | -1.383591147 |
| GIMAP4 | 1.292982727 |
| TMEM144 | 1.000952987 |
| DRAM1 | 1.292002878 |
| HEMGN | 1.413627806 |
| YOD1 | -1.023327321 |
| BATF3 | 1.592959944 |
| CHD7 | -1.072792726 |
| ASF1B | 1.155973232 |
| ADAP2 | 1.408966595 |
| CISD1 | 1.054847733 |
| AJAP1 | -2.455315539 |
| PROS1 | 1.30505128 |

| | |
|---|---|
| KCNQ5 | -1.125529836 |
| SUCNR1 | 2.550151416 |
| SMCO4 | 1.399399119 |
| FAM20C | 1.084688504 |
| ADPRM | -1.095130183 |
| ACKR3 | 1.420428434 |
| PTAFR | 1.455872152 |
| C3orf14 | 1.445214135 |
| PTGS2 | 3.149964279 |
| PLEKHG5 | -1.266376519 |
| ERMN | -1.310843854 |
| PLEKHG1 | -1.003455874 |
| HOMEZ | 1.247026297 |
| ZDBF2 | -1.177201488 |
| CACHD1 | -1.621923591 |
| PTPRF | 1.035037638 |
| PTX3 | 3.502291539 |
| NECTIN1 | 1.463718318 |
| MS4A7 | 1.214919032 |
| NLRC4 | 1.198012995 |
| RGS16 | 1.848228344 |
| RNASE2 | 1.272653977 |
| RNASE6 | 1.124579153 |
| BACH2 | -1.129155563 |
| MBOAT4 | -1.130880247 |
| RRM2 | 1.206880385 |
| SORT1 | 1.185339597 |
| S100A8 | 1.055007327 |
| S100A9 | 1.132073067 |
| S100A12 | 1.152609191 |
| S100B | -2.705212406 |
| S100P | -2.467313189 |
| SCN4A | -1.753502777 |
| CCL2 | 5.397111847 |
| CCL3 | 3.378039555 |
| CCL4 | 2.606968145 |
| CCL20 | 7.559392562 |
| SDC4 | 1.437066277 |
| SECTM1 | 1.268529259 |
| TSPYL2 | -1.095580484 |
| C19orf33 | 1.683133857 |
| LIPN | 1.070671976 |
| RNF222 | -2.157529331 |
| BLVRA | 1.166914428 |
| MTARC1 | 1.089643231 |
| NCF1 | 1.118024437 |
| H3C13 | -2.0959699 |
| TMEM183B | 1.629122699 |
| SLC6A12 | 1.789033244 |
| ZSCAN18 | -1.029432732 |
| LYNX1 | 1.202867375 |
| SIGLEC1 | 3.109516108 |
| SOX5 | -1.738516359 |
| SPINT1 | 1.069724546 |
| BST2 | 1.199302441 |
| ZEB1 | -1.008018059 |
| TCN2 | 1.786421537 |
| TGM3 | -3.899366419 |
| THBD | 2.564816828 |
| THBS1 | 2.948441008 |
| THBS2 | 1.504879942 |
| KLF10 | 1.065428456 |
| TIMP1 | 1.043205081 |

| | |
|---|---|
| TLR4 | 1.684465604 |
| SERPING1 | 2.735408671 |
| TLR5 | 1.084810542 |
| C1QA | 1.855918923 |
| CLEC3B | 1.023411778 |
| TNF | 3.011188269 |
| C1QB | 2.260772223 |
| TNFRSF1A | 1.087747482 |
| C1QC | 2.095058865 |
| TNNT3 | -1.223837557 |
| TNS1 | 1.002044437 |
| C2 | 1.339360544 |
| C3AR1 | 2.703762344 |
| TUB | -1.113975067 |
| C5AR1 | 1.175340326 |
| CCR2 | 2.642331429 |
| PLIN4 | -1.572898456 |
| LOC730098 | -1.02434862 |
| KDM6A | -1.144075514 |
| WARS1 | 1.026081841 |
| WNT7A | -1.076661829 |
| CA1 | 2.088689999 |
| CA4 | -4.11475057 |
| MKRN3 | -2.101611295 |
| ZKSCAN8 | -1.039134844 |
| ZXDA | -1.001744575 |
| RNF103 | -1.082922837 |
| TMEM38A | 1.217459486 |
| PRRG4 | 1.07900236 |
| MMP28 | -1.460667158 |
| LILRA6 | 1.22134678 |
| ZBED2 | -1.187531006 |
| TMEM231 | -1.197605117 |
| TNFAIP8L2 | 1.407306422 |
| EPHX3 | 1.651424197 |
| PLBD1 | 1.07045868 |
| CCNP | -1.264603397 |
| CALD1 | 1.260684009 |
| MYCT1 | 1.17663794 |
| WNT5B | 2.769727725 |
| CAMK4 | -1.278154701 |
| MKKS | 1.160856478 |
| GATD3A | -1.427658093 |
| DYSF | 1.081843557 |
| H4C9 | -2.607753944 |
| H2AC20 | -1.297322028 |
| H2BC8 | -2.210529187 |
| H2BC14 | -4.018349533 |
| H2BC7 | -2.042098844 |
| H3C4 | -1.469166541 |
| OBSCN | -1.23683662 |
| SYDE2 | -1.828221401 |
| C9orf64 | 1.370092652 |
| C15orf48 | 3.565767422 |
| DOC2A | 1.322038516 |
| GON7 | 1.066742501 |
| CARD6 | 1.456236721 |
| BEX2 | -1.037801208 |
| TXNDC17 | 1.125335781 |
| HAVCR2 | 1.181041305 |
| TMEM60 | 1.238192308 |
| PAQR8 | 1.615650338 |
| LGALS12 | 1.144823799 |

| | |
|---|---|
| APOL1 | 1.01722037 |
| KMO | 1.067098698 |
| OASL | 2.112088082 |
| SOCS1 | 1.132531012 |
| CBR1 | 1.029921318 |
| TNFSF10 | 2.019368167 |
| TNFRSF10D | 1.164593664 |
| INPP4B | -1.061650204 |
| IER3 | 2.649732906 |
| CACNA1I | -1.097663116 |
| STBD1 | 1.15394905 |
| TMEM250 | 1.11215406 |
| RHBDL1 | -1.180017931 |
| LDB2 | -1.558975355 |
| RSAD2 | 3.071259693 |
| NEXN | 1.441436962 |
| UBE2L6 | 1.023367584 |
| CD9 | 1.106897308 |
| CD14 | 1.201372052 |
| OTOF | 2.423310785 |
| ACVRL1 | 1.967357646 |
| MUC16 | -1.521697933 |
| TNFRSF8 | 1.933937298 |
| CHST2 | 1.089517615 |
| HOMER3 | 1.040097902 |
| CCL4L2 | 3.631353852 |
| ISG15 | 2.200376944 |
| SDC3 | 1.437201301 |
| GPRASP1 | -1.272354601 |
| CD151 | 1.058677276 |
| CDC20 | 1.316714436 |
| MAFB | 2.127975933 |
| HS3ST3A1 | -3.827949216 |

**Table S2.**

| **Compounds** | **Log2FC** |
|---|---|
| Androstanedione | 3.0348225039652266 |
| Arg Thr Ile | -2.7606471896012765 |
| 9,10-DiHOME | -2.289095390293185 |
| 6Naltrexol | 5.692021875955452 |
| (6E,8E,10E,12E,14E,16E,18E,20E,22E,26E)-31-methoxy-2,6,10,14,19,23,27,31-octamethyldotriaconta-2,6,8,10,12,14,16,18,20,22,26-undecaene | -4.5315762702122715 |
| (1R,16Z,27Z,29Z)-1,18-dihydroxy-12-[1-(4-hydroxy-3-methoxycyclohexyl)propan-2-yl]-19,31-dimethoxy-15,17,21,23,30,36-hexamethyl-11,25,37-trioxa-4-azatetracyclo[31.3.1.0(2),(2)]heptatriaconta-16,27,29-triene-2,3,10,14,20-pentone | -4.508390229088704 |
| Norpropoxyphene | 5.537974340842302 |
| 1-Naphthalenesulfonic acid, 8-(phenylamino)- | 8.443881962888032 |
| Val Val Val Gly Gly | 2.2469670068242147 |
| Phe Leu Val Gly Gly | -2.9492672380080487 |
| Gly Lys Thr Ser Glu | -6.584233249123049 |
| PE-NMe(20:0/22:2(13Z,16Z)) | 5.315134295673473 |
| 4-Hydroxy-3-methoxy-2,10-bisaboladien-9-one | 3.4580614034551465 |
| 17beta-Hydroxy-7alpha-methylandrost-1,4-diene-3-one;1-Dehydro-7alpha-methyltestosterone | 4.9015099501524855 |

| | |
|---|---|
| Val Arg Ala Asp Leu | -3.8929840029498513 |
| PE-NMe(15:0/24:1(15Z)) | -4.504501300968029 |
| Neostigmine | 4.489398970190744 |
| Phe Tyr Lys Arg | -2.7700183646700984 |
| Tyr His Arg Arg | -3.2213486374206792 |
| PE-NMe(16:1(9Z)/16:1(9Z)) | 3.561329854345989 |
| TG(16:0/20:3(5Z,8Z,11Z)/14:1(9Z)) | -4.296350573736757 |
| 5(S),6(S)-DiHETE | 2.338071264116127 |
| Lys Leu His Val Asp | -3.660418519084225 |
| Gln Phe Leu Glu | 3.0013510619415973 |
| Arg Val Ser | -3.044100907259353 |
| Tetranactin | 5.621173584899262 |
| Alangicine | -7.319870226389567 |
| DG(i-22:0/10:0/0:0) | 3.9510301027971413 |
| R.g.-Keto III | 3.7168705404058997 |
| Tributyl phosphate | 1.9456158097937024 |
| trans-2-Dodecenal | -1.8217946698117733 |
| Asp Lys Arg Glu Lys | -2.6191912737541223 |
| Rifabutin | -2.73655283502538 |
| Phenazocine | 5.5825041446389045 |
| Lys Thr Arg Val Ile | 2.0884465775508603 |
| Azaspiracid | -4.2322931435143785 |
| Prostaglandin D2 ethanolamide | 5.7359434538523075 |
| Santiaguine | 3.4295391329929377 |
| PC(16:0/20:1(11Z)) | 5.568455419852153 |
| 9(10)-EpOME | -3.1721163762078612 |
| 2-Linoleoylglycerol | 2.3874135556099736 |
| Glu Asp Arg Arg | -3.8609595444699054 |
| Asp Phe Gln Arg | -3.0007588347032286 |
| Tyr Leu Ala Lys | -2.5315277494095367 |
| PE-NMe2(15:0/14:1(9Z)) | 3.3544099689948084 |
| Ser Leu Leu Ser Phe | 6.1944023834556186 |
| PA(18:1(9Z)/20:3(8Z,11Z,14Z)) | 4.767880398748027 |
| PE(16:0/14:1(9Z)) | 2.0170886020056056 |
| Capsaicin | 2.30864430951231 |
| L,L-Cyclo(leucylprolyl) | 4.739262968764908 |
| Carnosifloside I | -5.130377001355009 |
| 1-(4-Aminobutyl)urea | 3.481412053167797 |
| Pectenotoxin 1;PTX 1 | -7.264268223483169 |
| Leu Glu Val Val Lys | 2.2301246505818075 |
| Val Ala Gly Val Ala | 5.568559524514859 |
| Cauloside C | -2.5842937989221584 |
| Docosahexaenoylserotonin | 2.4753643746311083 |
| Mahanimbinol | 3.8091194007750553 |

| | |
|---|---|
| Solanine; (2S,3R,4R,5R,6S)-2-[(2R,3R,4S,5S,6R)-5-Hydroxy-6-(hydroxymethyl)-2-[[(1S,2S,7S,10R,11S,14S,15R,16S,17S,20S,23S)-10,14,16,20-tetramethyl-22-azahexacyclo[12.10.0.02,11.05,10.015,23.017,22]tetracos-4-en-7-yl]oxy]-4-[(2S,3R,4S,5S,6R)-3,4,5-trihydroxy-6-(hydroxymethyl)oxan-2-yl]oxyoxan-3-yl]oxy-6-methyloxane-3,4,5-triol | 3.1652393883638053 |
| Dethiobiotin | 3.2055513056860523 |
| Pyrrolidine, 2-(diphenylmethyl)-, (2S)- | 3.5947894035762267 |
| Furmecyclox | 3.7315285935682345 |
| Pronetalol | -3.1271138520941903 |
| Tragopogonsaponin L | -5.807150113536363 |
| Bupranolol | 3.3878336754529332 |
| Phe Leu Leu Val Phe | 8.926798493385848 |
| Tyrosyl-Isoleucine | 1.2995881917821346 |
| Gly Glu Gly Phe Lys | -1.0165244502121031 |
| Oleic Acid-biotin | 1.7764954041792618 |
| Myristic acid | 1.5045927351078232 |
| Leu-Val | 1.1426822015469464 |
| LPC(0:0/14:0) | -1.2991204460111367 |
| 8-[(1R,2R)-3-Oxo-2-{(Z)-pent-2-enyl}cyclopentyl]octanoate;OPC-8:0 | 1.9446304141557915 |
| 5S,8R-DiHODE;(5S,8R)-DiHODE;(5S,8R,9Z,12Z)-5,8-Dihydroxyoctadeca-9,12-dienoate | 2.834294722761636 |
| 9E,11E-Octadecadienoic acid | 2.6718274583841555 |
| PC(20:0/22:6(4Z,7Z,10Z,13Z,16Z,19Z)) | -1.147111208979082 |
| Lys Phe Phe | 4.586458139910339 |
| PE-NMe(16:1(9Z)/22:5(4Z,7Z,10Z,13Z,16Z)) | 2.280997099979873 |
| Dihomo- -Linolenoyl PAF C-16 | 1.149926335182382 |
| Guanosine | 3.5789641880543663 |
| 11α-Hydroxytestosterone | 1.1142374145714051 |
| Megestrol acetate | 2.805814450414216 |
| 10,16-Dihydroxyhexadecanoic acid | 1.0076524709415735 |
| PS(16:1(9Z)/20:3(8Z,11Z,14Z)) | 3.1841515673367544 |
| 5,6-DiHETrE | 2.376921012009112 |
| (5Z,8Z,10E,14Z)-12-hydroxyicosa-5,8,10,14-tetraenoic acid | -1.962592840269132 |
| (3R,2'S)-Myxol 2'-alpha-L-fucoside | 4.5599448883084905 |
| 16-Oxo-palmitate | 2.0257829938224905 |
| LPC(16:2/0:0) | -1.3659098244895165 |
| 2-O-ethyl PAF C-16 | 1.250692710703627 |
| Sancuso | -4.74039136775015 |
| Pipericine | -1.2848870307440707 |
| Endomorphin-2 | -1.1494854118375222 |
| PA(16:1(9Z)/18:4(6Z,9Z,12Z,15Z)) | 2.0560656117058698 |
| PE(20:4(5Z,8Z,11Z,14Z)/14:1(9Z)) | 4.5612276752853465 |
| omega-3 Arachidonic Acid | 1.121910816751785 |
| PC(20:1(11Z)/18:1(9Z)) | 1.4258640571506132 |
| PC(16:0/20:3(5Z,8Z,11Z)) | 1.514843212610573 |
| Dodecahydro-8''-hydroxy-1'',2'(5'h)-furan-5',5''-[5h-1,4a](methanoxymethano)naphthalen]-2,9''-dione | 1.3839540289982801 |
| 3'-O-Methylbatatasin III | -1.3392178413030111 |

| | |
|---|---|
| Vindoline | 2.017305082637744 |
| DG(20:5(5Z,8Z,11Z,14Z,17Z)/22:2(13Z,16Z)/0:0) | 4.151030917138642 |
| LysoPE(0:0/20:1(11Z)) | 2.125831959102595 |
| Cymathere lactone | 1.185920074236886 |
| Leu Gln Arg Arg | 1.7510237807561275 |
| Rhodopinal | 4.757518226841402 |
| Octanal | -2.5345553975381807 |
| 19-Hydroxytestosterone | 2.565562952740568 |
| 17-phenyl trinor PGF2 cyclohexyl amide | -1.1921290642737776 |
| Thr Ile Pro Ile Thr | 1.3658500833659657 |
| Lys Ile Asp | 1.609488502888964 |
| DG(18:2(9Z,12Z)/14:1(9Z)/0:0) | -1.765910923543218 |
| Calpeptin | -1.4717782278175078 |
| Glu Lys His Cys Arg | 1.4086057135581749 |
| Asn Ala Leu Ala His | 1.3689304517542005 |
| Tyr Lys Val Glu Ile | -1.007996074347239 |
| Tryptophyl-Asparagine | 3.51111465870982 |
| Palmitelaidic acid | 2.8341550317234665 |
| PC(12:0/12:0) | 1.0182132029768227 |
| 13-Hydroxy-9Z,11E-octadecadienoic acid | 3.124321290565544 |
| Tautomycin | -1.2977982522513718 |
| Met Lys Lys | -1.9008553097447607 |
| DG(20:5(5Z,8Z,11Z,14Z,17Z)/14:1(9Z)/0:0) | 2.7019997044539545 |
| PE(16:0/22:0) | 1.739288021486381 |
| Prostaglandin H2 | -2.4818958806082168 |
| PE-NMe(15:0/20:5(5Z,8Z,11Z,14Z,17Z)) | 2.390092470345604 |
| gamma-Linolenic acid | 2.002933720081879 |
| LysoPE(0:0/18:2(9Z,12Z)) | -3.1168306679072355 |
| MG(0:0/18:3(9Z,12Z,15Z)/0:0) | 3.710740893760063 |
| 5,6-EET | 1.1764631412164517 |
| 17beta-Nitro-5alpha-androstane | 3.514411822211203 |
| LPE(18:0/0:0) | -1.4938195835615329 |
| 1-Palmitoyl-sn-glycero-3-phosphocholine | 2.3906097564086672 |
| Linolenic acid | 1.3806842836568938 |
| [(2S)-2-[(6Z,9Z,12Z,15Z)-octadeca-6,9,12,15-tetraenoyl]oxy-3-[(Z)-tetradec-9-enoyl]oxypropyl] (7Z,10Z,13Z,16Z)-docosa-7,10,13,16-tetraenoate | -1.9637240038317159 |
| Inosine | 3.0652001907281035 |
| Cyanidin 3-rutinoside 5-glucoside chloride | 1.7902610559391066 |
| Pro Glu Lys | 1.7429594108102404 |
| PE-NMe(18:3(6Z,9Z,12Z)/20:5(5Z,8Z,11Z,14Z,17Z)) | -2.5409699096306104 |
| Oleate | 2.0817435865991727 |
| Iomeprol | -1.1745118686259417 |
| Hydroxyspirilloxanthin;Monodemethyl spirilloxanthin | -1.079965061917994 |
| Ile Phe Arg Lys | 1.57630926340733 |

| | |
|---|---|
| Phe Asn Ile Glu | -1.3114973748030223 |
| TG(18:2(9Z,12Z)/20:5(5Z,8Z,11Z,14Z,17Z)/18:4(6Z,9Z,12Z,15Z)) | 1.0833875688155437 |
| P-Aminobenzoate | 3.0318847212206226 |
| PC(16:1(9Z)/20:0) | 2.497055643745587 |
| SM(d18:1/16:0) | -2.8049866327012696 |
| PE-NMe(15:0/18:3(6Z,9Z,12Z)) | -5.234194750863409 |
| Ecdysone | 2.047581156414989 |
| DG(20:0/18:3(6Z,9Z,12Z)/0:0) | -2.268112074335004 |
| Tricyclazole | -1.991541923611841 |
| Phe Ile Val Gly Asp | 1.2951381213566788 |
| Sitagliptin | -1.0822908874564396 |
| DG(18:0/20:3(5Z,8Z,11Z)/0:0) | 3.9353219482694817 |
| N-Palmitoyltryptamine | -1.881141429809687 |
| 22-Angeloyltheasapogenol A | 6.554445792388402 |
| MG(18:1(9Z)/0:0/0:0) | 4.922655144479952 |
| N-Tetradecanoyl-L-homoserine lactone | 1.7930726174500533 |
| Leupeptin | 3.7958579824879615 |
| His Val Leu Lys | -1.001550295532133 |
| Bufalin | 1.8858439172293595 |
| Phe Phe Val | 4.103290408736794 |
| Aspartyl-Leucine | -1.5201323204485555 |
| Ser His Val Lys | 1.04783022544524 |
| (±)-11-Hydroxy-.DELTA.9-tetrahydrocannabinol | 1.4518064756634428 |
| 2-Arachidonoylglycerol | 1.4138047379436045 |
| N-Docosanoyl-4-sphingenyl-1-O-phosphorylcholine | 3.683990933546312 |
| Janthitrem C | 3.3819702657951587 |
| Manzamine A;Keramamine A | 1.7801580547717568 |
| Leu Tyr Lys Glu | 1.838816778264554 |
| PE-NMe(14:1(9Z)/18:4(6Z,9Z,12Z,15Z)) | -2.3093359489074667 |
| 1,2-Distearoyl-sn-glycero-3-phospho-L-serine | 1.6411560342158082 |
| Propanoic acid, 2-[[1-[3-[4-([1,1'-biphenyl]-4-ylcarbonyl)-2-propylphenoxy] propyl]-1,2,3,4-tetrahydro-5-quinolinyl]oxy]-2-methyl- | 4.174361280483712 |
| PA(20:1(11Z)/18:1(11Z)) | 5.048247023172331 |
| (-)-Malyngolide | 2.9727876156413275 |
| Ile Ser Val Asp | 1.7025241260067785 |
| PA(20:0/22:2(13Z,16Z)) | -3.9136359054956302 |
| Actinonin | 1.027893234169315 |
| Ile Leu Leu Gly Ala | 1.745377684671659 |
| Phe-Phe | -1.08039378628695 |
| 4-methyl-2-oxovaleric acid | -2.707167462734146 |
| Linoleoylcarnitine | -1.0363390674562756 |
| Asp Gln Phe Arg | 1.4371966276058254 |
| Clomipramine | -1.0107572869516392 |
| Lys Leu Arg Val Asp | -2.9777735868623374 |

| | |
|---|---|
| Doramapimod | 2.154638408088186 |
| PE-NMe2(16:0/15:0) | 5.819783250590175 |
| Arg Thr Ala Arg | -1.251291056226184 |
| Leukotriene D4 methyl ester | 1.7828725495082178 |
| 1-Palmitoyl-2-myristoyl-sn-glycero-3-phosphocholine | -3.0151394938509823 |
| Gly Leu Arg Val Phe | 1.5866622980287441 |
| PE-NMe(20:0/24:0) | 1.164107346477916 |
| 18-Hydroxyoleate | 1.925406680521732 |
| LPC(20:4/0:0) | 1.8927632173783884 |
| L-Palmitoylcarnitine | -1.0714127210517868 |
| Met Trp Met | 2.4941067395432115 |
| Val-Val-Val | -2.6228651861860897 |
| Leu Lys His Glu | 1.94838491771032 |
| cis-9-Hexadecenoic acid | 1.3450339074735709 |
| 15-Keto-Prostaglandin F2a | -4.788482464062663 |
| Benzyl butyl phthalate | 2.8481733667323006 |
| PA(16:1(9Z)/20:3(5Z,8Z,11Z)) | -8.547651885797595 |
| PS(18:1(9Z)/18:2(9Z,12Z)) | -6.812546010070611 |
| Pro-Thr | 1.0331153861534288 |
| PA(a-25:0/i-12:0) | 5.10042837900699 |
| Phe-Ser | 1.1274751859293624 |
| Benzylamine | 1.7631688769325755 |
| Nomilin | 1.3133334374589345 |
| MG(18:0/0:0/0:0) | 3.266130407129221 |
| Pro-Val | -1.0693117149259417 |
| Deoxybrevianamide E | -1.418940418647504 |
| DG(18:4(6Z,9Z,12Z,15Z)/20:4(8Z,11Z,14Z,17Z)/0:0) | -5.999514752869394 |
| TG(20:0/10:0/10:0) | -5.7495397330401286 |
| Ile Glu Leu Lys | -1.6543192805359042 |
| LPE(20:4/0:0) | 1.7449251065505722 |
| Ser Asp Ile Glu Val | 1.085277783344244 |
| MG(22:5/0:0/0:0) | 3.002783471690869 |
| MG(0:0/22:6/0:0) | 4.0703602649541 |
| PA(18:1(9Z)/18:2(9Z,12Z)) | -7.8139942936004925 |
| Tryprostatin b | -1.3692202483205973 |
| Thr Leu Pro Pro Gly | -1.2937911637901165 |
| Ser Leu Pro Tyr Ala | 1.8824845081402757 |
| 13-Demethyl tacrolimus | -9.458787286353108 |
| Vaccenic acid | 2.100532716995681 |
| PE(14:1(9Z)/15:0) | 4.176094324571975 |
| 1,2-Di-(9Z-tetradecenoyl)-sn-glycero-3-phosphocholine | -1.4870333563654732 |
| Hexadecanal | 2.7468647185389607 |
| PA(18:1(11Z)/22:5(4Z,7Z,10Z,13Z,16Z)) | -6.824597022554396 |
| Carindone | 3.0109724608712845 |

| | |
|---|---|
| TG(15:0/14:1(9Z)/16:1(9Z)) | -8.636946794515708 |
| Avermectin A1a aglycone | -4.845075635027748 |
| PE-NMe(14:0/24:1(15Z)) | -1.1821925121194898 |
| Oligomycin C | -1.4238903574726778 |
| MG(20:4/0:0/0:0) | 4.5316868177505745 |
| Pseudopelletierine | 1.4162951281985363 |
| DG(18:1(9Z)/20:0/0:0) | -5.960943668301993 |
| Thiamine monophosphate | 3.932415137570932 |
| trans-Vaccenic acid | 1.8207145722894071 |
| LPC(0:0/20:5) | 1.6256112677901229 |
| Westiellamide | -2.7676065654868065 |
| PC(14:0/18:1(11Z)) | -5.413667626474752 |
| DG(20:5(5Z,8Z,11Z,14Z,17Z)/20:2(11Z,14Z)/0:0) | -5.440884444561534 |
| Lys Tyr Thr Arg | -1.3883182186401493 |
| PE(14:1(9Z)/20:2(11Z,14Z)) | 6.02985974396448 |
| DG(18:3(6Z,9Z,12Z)/18:4(6Z,9Z,12Z,15Z)/0:0) | -5.7533627893811286 |
| Tetranor 12-HETE | 3.6383360310621313 |
| Gymnemic acid I | -5.26866765069996 |
| 1-Arachidonoylglycerol | 3.5146680804225867 |
| Ile Ala Leu Arg | 1.6619819608051831 |
| Pirimicarb | -2.971514824854605 |
| Lysope 18:1 | 1.6407765391421028 |
| Bolegrevilol | 2.0777482764787747 |
| Hexaethylene glycol | -4.291566462388877 |
| PE-NMe(15:0/16:0) | -5.115403852335408 |
| Ala Lys Gly Arg Val | 3.8155754288625734 |
| Vildagliptin | -1.5717972921339407 |
| 12,13-DiHOME | -4.76811853771425 |
| PA(18:3(6Z,9Z,12Z)/18:1(11Z)) | -6.935241277356255 |
| (32RS)-Rapamycin | -1.9371439120159768 |
| Thr Leu Thr Gly Lys | -3.6952898655259423 |
| PC(14:0/15:0) | -3.6326074714069496 |
| 1-O-Hexadecyl-2-O-methyl-sn-glyceryl-3-phosphorylcholine | -3.3023526303436066 |
| MG(0:0/18:1(9Z)/0:0) | 3.7413718743669655 |
| [(2S)-3-dodecanoyloxy-2-tetradecanoyloxypropyl] tetradecanoate | -7.245834206048131 |
| TG(18:0/18:3(6Z,9Z,12Z)/14:1(9Z)) | -5.609187917362333 |
| Asp Pro Lys Ile Ala | -2.6679190761123177 |
| METHACHOLINE | -1.405404132922476 |
| Benzene | -1.5942164023697656 |
| Glycidyl oleate | 3.3332539803586574 |
| Val Ile Lys Asp | -4.206691249341199 |
| Dodecyl glucoside | -7.189704817873306 |
| Prenoxdiazine | -2.1757160901412456 |
| Ala Ser Lys | 1.3031549279465768 |

| | |
|---|---|
| azido-FTY720 | -6.270753332122999 |
| DG(18:4(6Z,9Z,12Z,15Z)/18:4(6Z,9Z,12Z,15Z)/0:0) | -1.2643823040282351 |
| Arg Gln Arg | 1.2759326258923809 |
| Tenovin-6 | -5.343369146414876 |
| Ala Phe Ile | -5.517086318459692 |
| Mepiprazole | -4.142497916892194 |
| Leu Gly Asp Val Ile | -3.0438203574536447 |
| Acitretin | -5.729971458528872 |
| Val Ile Asp Lys | 4.0159763104298785 |
| Asn Gln Gln | -2.196633551230084 |
| Isouron | -3.193008395246259 |
| Lynestrenol | 1.276074290229854 |
| 17(R)-Hydroxydocosahexaenoic acid | -1.8683148259707791 |
| His Lys His | 1.4271631674657648 |
| Mancinellin | -8.631666505665518 |
| Jasmonic acid | -2.4106949402597144 |
| Dodecylbenzenesulfonic acid | -5.557187316753964 |
| Ala Thr Ile Lys | -7.391026560022255 |
| Adlupone | -4.591881310560409 |
| Carbaprostacyclin-biotin | -6.534584611279284 |
| MG(18:2/0:0/0:0) | 2.9026463022120863 |
| PA(20:0/20:3(5Z,8Z,11Z)) | -6.356700659482348 |
| Val Ile Leu Asp | -6.261719455277762 |
| Arg Ile Val Leu Val | -7.1527086101175525 |
| Anthranoyllycoctonine | -5.578829934419524 |
| DG(20:0/10:0/0:0) | 4.1582649890577645 |
| Ginsenoside Rg2 | -3.2512333654711667 |
| Eudesobovatol B | -3.803222995923794 |
| PE-NMe(14:0/20:1(11Z)) | -6.163950671991416 |
| Butanoic acid, 4-((1,2-dioxohexadecyl)amino)-, ethyl ester | -2.789308523113801 |
| PA(16:1(9Z)/22:0) | -2.4524318031302577 |
| PE-NMe(16:0/15:0) | -3.819981348332069 |
| PA(18:0/22:6(4Z,7Z,10Z,13Z,16Z,19Z)) | -8.676858781360359 |
| PC(15:0/14:0) | -8.58027217786435 |
| 16,16-Dimethylprostaglandin E1 | -4.1840415924819885 |
| Diacetyl-10-gingerdiol | 2.7201717234144605 |
| Dipyridamole | -5.159390874218862 |
| 17-Octadecynoic acid | 2.5095523967810958 |
| Dexamethasone | -6.002940976585058 |
| PC(15:0/20:2(11Z,14Z)) | -3.1761370812276697 |
| Lys Phe Leu Glu | -5.618860684697969 |
| His Leu Tyr Lys | -3.4778050965063567 |
| PE-NMe(14:1(9Z)/16:1(9Z)) | -2.021494644410344 |
| Arg Phe Met | -3.2591879879976564 |

| | |
|---|---|
| 1-Octadecyl lysophosphatidic acid | -3.4086405930031645 |
| Sirolimus | -3.612353348534436 |
| PA(18:3(6Z,9Z,12Z)/18:1(9Z)) | -6.181015445143019 |
| 1-(1,2-Dihexanoylphosphatidyl)inositol-3,4-bisphosphate | -9.542792840620704 |
| MEFEXAMIDE | 3.3167140412650378 |
| (3S)-6-[(1E,3E,5E,7E,9E,11E,13E,15E,17E)-18-[(4R)-4-hydroxy-2,6,6-trimethylcyclohexen-1-yl]-3,7,12,16-tetramethyloctadeca-1,3,5,7,9,11,13,15,17-nonaenyl]-1,5,5-trimethyl-7-oxabicyclo[4.1.0]heptan-3-ol | -6.373874205265956 |
| Geissospermine | -3.2046787199680407 |
| Simvastatin | -3.8148808870159048 |
| Arg Ser Ser | -5.2408628629658836 |
| Gly Leu Arg Asn Gln | -3.0202903501615563 |
| Tetraethylene glycol | -1.3974343165308407 |
| (2S)-2-[[2-(1H-indol-3-yl)acetyl]amino]-4-methylpentanoic acid | -1.1608822399001242 |
| MG(18:1/0:0/0:0) | 3.3702197077976934 |
| Inosine 5'-monophosphate | -1.266915900996481 |
| 4,5-Epoxy-7Z,10Z,13Z,16Z,19Z-docosapentaenoic acid, methyl ester | 1.4075159715070935 |
| Isoprenaline | 4.802409348728858 |
| Val Pro Glu Pro Lys | -5.931585375553185 |
| 11-Cis-Retinol | -1.4537375756050608 |
| Thr Asn Asp Lys | -6.462994193012447 |
| PE-NMe2(18:0/22:4(7Z,10Z,13Z,16Z)) | 3.4812141272130037 |
| Flurandrenolide | -7.05079335132944 |
